# ﻿Novel endophytic fungi from Pinus: introducing three new genera and six new species within *Capnodiales* (*Ascomycota*, *Dothideomycetes*) sensu lato

**DOI:** 10.3897/imafungus.16.175931

**Published:** 2025-12-11

**Authors:** Xiu-Yu Zhang, Qiu-Yue Zhang, De-Wei Li, Jing-Zu Sun, Ben Fan

**Affiliations:** 1 College of Forestry and Grassland, Nanjing Forestry University, Nanjing, Jiangsu 210037, China Nanjing Forestry University Nanjing China; 2 The Connecticut Agricultural Experiment Station Valley Laboratory, Windsor, CT 06095, USA The Connecticut Agricultural Experiment Station Valley Laboratory Windsor United States of America; 3 Laboratory of Microbiome & Microecological Technology, Institute of Microbiology, Chinese Academy of Sciences, Beijing 100101, China Chinese Academy of Sciences Beijing China

**Keywords:** *
Capnodiales
*, endophytic fungi, new taxa, phylogeny, pine, taxonomy

## Abstract

*Capnodiales* s. lat., the second largest order within the *Dothideomycetes*, comprises a highly diverse group of species exhibiting considerable morphological and phylogenetic diversity. In this study, we examined more than 800 fungal isolates from shoots of three species of healthy pine trees (*Pinus densiflora* Siebold & Zucc., *P. thunbergii* Parl., and *P. elliottii* Engelm.) in China, leading to the establishment of several novel taxa with *Capnodiales* s. lat.. The species were identified through morphological characterisation and phylogenetic analyses based on concatenated sequence of seven loci: the internal transcribed spacer (ITS) regions, the large subunit nuclear ribosomal RNA gene (nLSU), the RNA polymerase II subunit B (*RPB2*), the actin gene (*ACT*), the calmodulin gene (*CAL*), the ß-tubulin gene (*TUB*) and the elongation factor 1-alpha gene (*TEF1*). As a result, three novel genera—*Botryoconidia (Dissoconiaceae)*, *Longisporomyces (Extremaceae)*, and *Helianthoconium (Mycosphaerellaceae)* are proposed, along with nine novel species: *Botryoconidia globosus*, *Longisporomyces filisporum*, *Neocatenulostroma endophyticum*, *Rachicladosporium pennatum*, *Helianthoconium helianthosporum*, *Sphaerulina nanjingensis*, *Toxicocladosporium fusiforme*, *Zasmidium guttulatum*, and *Zasmidium longisporum*. Furthermore, we employed fossil calibrations to estimate divergence times of these taxa within *Capnodiales* s. lat. The results suggest that the crown age of *Capnodiales* was around 241.9 Mya (95% HPD: 239.23–243.18 Mya), in the Middle Triassic. The major subclades such as *Capnodiales* s. str., *Cladosporiales* and *Mycosphaerellales* began diversifying during the Jurassic, with crown ages estimated at 169.56 Mya, 148.60 Mya, 187.18 Mya, respectively. This study provides comprehensive descriptions of these new endophytic taxa from pine hosts, contributing to the taxonomic and systematic understanding of the *Capnodiales* s. lat.

## ﻿Introduction

The delineation of the order *Capnodiales* has undergone significant revisions over time. Historically, fungal classification relied heavily on morphology, particularly characteristics of ascomata, ascospores, and asexual states (conidiomata and conidia). The foundation lineage of *Capnodiales* was established by Everett S. Luttrell (Luttrell 1955), based on a multilocus phylogeny with the presence of ostiolar periphyses as a synapomorphic feature (Lumbsch and Lindemuth 2001). However, subsequent phylogenetic analyses using molecular data revealed the deeper complexities of this order (Luttrell 1955; Lumbsch and Lindemuth 2001). Molecular phylogenetics, involving key gene markers such as the internal transcribed spacer region (ITS), large subunit ribosomal RNA (nLSU), small subunit ribosomal RNA (SSU), translation elongation factor 1-α (*TEF1*), and beta-tubulin (*TUB*), demonstrated that *Capnodiales* sensu lato (s. lat.) is polyphyletic, with lineages distributed across *Dothideomycetes* and *Eurotiomycetes* (Hunter et al. 2006; Crous et al. 2007a; Crous et al. 2009c; Crous et al. 2009d; Hyde et al. 2024). While initial broad concepts were challenged by these modern classification methods, leading to proposals for splitting of *Capnodiales*, Schoch et al. (2006), Schoch and Grube (2015), and Hongsanan et al. (2020) advocated for retaining a larger, cohesive *Capnodiales* s. lat. unit encompassing 19 families. They emphasised molecular support for its integrity despite ecological diversity. However, the comprehensive studies by Crous (2009), Abdollahzadeh et al. (2020), and Hyde et al. (2024) necessitated a substantial reorganisation of *Capnodiales*. Abdollahzadeh et al. (2020) explicitly demonstrated the polyphyly of *Capnodiales* s. lat., resulting in the resurrection of *Mycosphaerellales* and the introduction of five new orders (*Cladosporiales*, *Comminutisporales*, *Neophaeothecales*, *Phaeothecales*, *Racodiales*). Building on this, Hyde et al. (2024) further refined the concept based on combined analyses of multi-locus phylogeny and morphology, reorganising the order *Capnodialessensu stricto* (s. str.) to comprise seven families: *Antennulariellaceae* Woron., *Capnodiaceae* Höhn. ex Theiss., *Euantennariaceae* S. Hughes & Corlett ex S. Hughes, *Johansoniaceae* Doilom, *Neoantennariellaceae* Abdollahz. & Crous, *Piedraiaceae* Viégas ex Cif., Bat. & S. Camposa, Phookamsak & K.D. Hyde, *Readerielliopsidaceae* Abdollahz. & Crous.

Ecologically, members of *Capnodiales* s. lat. exhibit remarkable versatility and global distribution. Encompassing a wide array of members with various lifestyles, including saprobes, plant and human pathogens, mycoparasites, lichenized fungi, and epiphytic sooty molds, *Capnodiales* s. lat. has become the second largest order within *Dothideomycetes* (Hughes 1976; Aptroot 2006; Schoch et al. 2006; Crous et al. 2009c; Agut 2010). Many species are ubiquitous, found on diverse substrates across various climates worldwide, while others display host or substrate specificity. Using ancestral state reconstruction, Abdollahzadeh et al. (2020) identified saprotrophy as the ancestral lifestyle and determined that multiple transitions have occurred towards parasitism, lichenization, and endophytism.​

The ecological relationship between lineages within *Capnodiales* s. lat. and coniferous hosts, particularly *Pinus* (pine) species, has been recorded. Members of this order, especially those in the families *Mycosphaerellaceae* (now elevated to *Mycosphaerellales*), include well-known pathogens that cause diseases like Dothistroma needle blight and brown spot needle blight on pines (Crous et al. 2007a; Crous et al. 2009c; Dobbs et al. 2024). Additionally, diverse endophytic fungi affiliated with *Capnodiales* s. lat. communities have been detected within healthy pine tissues, where they reside asymptomatically and are increasingly recognised for their potential roles in plant health and ecosystem functioning. However, compared to pathogenic or saprobic representatives, the diversity, phylogenetic positions, and ecological functions of the endophytes within *Capnodiales* s. lat. remain poorly understood. Systematic exploration of these endophytes in non-model hosts such as conifers is particularly limited. Although significant advances have been made, including divergence time estimates contributing to understanding the temporal diversification within *Capnodiales* s. lat. (Abdollahzadeh et al. 2020), the taxonomic boundaries and evolutionary trajectories of the group remain incompletely resolved.

This study investigated the diversity of endophytic fungi affiliated with *Capnodiales* s. lat., isolated from shoots of pine trees (*Pinus*) in China. We described novel taxa based on morphology and multi-locus phylogenies. Additionally, fossil calibrations were employed to estimate divergence times of key clades within *Capnodiales* s. lat., aiming at contributing to provide more insights into their evolutionary history and temporal diversification.​​​

## ﻿Materials and methods

### ﻿Isolation of endophytic fungi

Shoots of *P. densiflora* Siebold & Zucc., *P. thunbergii* Parl., and *P. elliottii* Engelm. were collected from Baima campus of Nanjing Forestry University, Nanjing, China, and packed in Ziploc bags. The first two species were introduced from Japan as pine wood nematode-resistant variety and have been grown in Nanjing for 20 years. The fresh samples were stored at 4 °C until fungal isolation. The isolation of endophytic fungi was performed following a previously described protocol (Arnold et al. 2007). Briefly, the needles were removed from the shoots, which were then cut into segments of approximately 1 cm. Subsequently, the samples were rinsed in tap water before being surface-sterilised by immersion in 75% ethanol for 1 min, followed by 1% sodium hypochlorite solution for 3 min, and another 75% ethanol treatment for 30 s. Finally, they were rinsed three times with sterile distilled water. The sterilized samples were ground using a sterile mortar and pestle, diluted with sterile water, and then plated onto the different medium agar. For each sample, at least three replicates were prepared per medium type. All plates were incubated at 25 °C for 3–7 days. Fungal strains were purified by streaking on malt extract agar (MEA) supplemented with 0.3 g/L streptomycin sulfate. In total, more than 800 endophytic fungal isolates were obtained. Preliminary identification based on NCBI BLAST searches of ITS sequences indicated that approximately 40 isolates potentially represent novel taxa with sequence similarities lower than 98%. Among them, 18 isolates belonged to the order *Capnodiales* (Table 1) and specified in the article.

**Table 1.** New taxa proposed in this study and their corresponding GenBank accession numbers.

Taxon	Accession number	Treehost	ITS	nLSU	*RPB2*	*TEF1*	*TUB*	*ACT*	*CAL*
*Botryoconidia globosus*	NF150 (T) = CGMCC 3.28956	*Pinus elliottii*	PX214404	PX214422	PX640838	PX640854	–	–	–
*B. globosus*	NF151	*P. elliottii*	PX214405	PX214423	PX640839	PX640855	–	–	–
*Longisporomyces filisporum*	NF595 (T) = CGMCC 3.29108	*P. densiflora*	PX214406	PX214424	PX640840	PX640856	–	–	–
*L. filisporum*	NF692	*P. densiflora*	PX214407	PX214425	PX640841	PX640857	–	–	–
*Neocatenulostroma endophyticum*	NF685 (T) = CGMCC 3.28957	*P. densiflora*	PX214408	PX214426	-	PX640858	–	PX640874	PX640876
*N. endophyticum*	NF690	*P. densiflora*	PX214409	PX214427	-	PX640859	–	PX640875	PX640877
*Rachicladosporium pennatum*	NF412 (T) = CGMCC 3.28959	*P. thunbergii*	PX214410	PX214428	PX640842	PX640860	–	–	–
*R. pennatum*	NF413	*P. thunbergii*	PX214411	PX214429	PX640843	PX640861	–	–	–
*R. pennatum*	NF416	*P. thunbergii*	PX214412	PX214430	PX640844	PX640862	–	–	–
*Helianthoconium helianthosporum*	NF684 (T)= CGMCC 3.28953	*P. densiflora*	PX214413	PX214431	PX640845	PX640863	–	–	–
*H. helianthosporum*	NF884	*P. densiflora*	PX214414	PX214432	PX640846	PX640864	–	–	–
*Sphaerulina nanjingensis*	NF651 (T) = CGMCC 3.28952	*P. densiflora*	PX214415	PX214433	PX640847	PX640865	PX640872	–	–
*S. nanjingensis*	NF851	*P. densiflora*	PX214416	PX214434	PX640848	PX640866	PX640873	–	–
*Toxicocladosporium fusiforme*	NF414 (T) = CGMCC 3.28958	*P. thunbergii*	PX214417	PX214435	PX640849	PX640867	–	–	–
*Zasmidium guttulatum*	NF649 (T) = CGMCC 3.28955	*P. densiflora*	PX214418	PX214436	PX640850	PX640868	–	–	–
*Z. guttulatum*	NF648	*P. densiflora*	PX214419	PX214437	PX640851	PX640869	–	–	–
*Z. longisporum*	NF622 (T) = CGMCC 3.28954	*P. densiflora*	PX214420	PX214438	PX640852	PX640870	–	–	–
*Z. longisporum*	NF822	*P. densiflora*	PX214421	PX214439	PX640853	PX640871	–	–	–

All fungal isolates were deposited in the Microbial Culture Collection of Nanjing Forestry University, Nanjing, Jiangsu, China (see Table 1). Holotype cultures of the new species described in this study were deposited in the China General Microbiological Culture Collection Center (CGMCC; http://www.cgmcc.net/english/catalogue.html), Beijing, China. The corresponding holotype specimens (dry cultures) were accessioned in the Herbarium Mycologicum Academiae Sinicae (HMAS), Beijing, China (Table 1).

### ﻿Media and growth condition

Different media were used for isolation of the endophytic fungi including 2% MEA medium (Malt Extract Agar: 20 g agar, 20 g malt extract, 1 L deionized water), Martin’s medium (20 g agar, 10 g glucose, 5 g peptone, 1 g K_2_HPO_4_, 0.5 g MgSO_4_·7H_2_O, 1 L deionized water), SNA medium (1.0 g KH_2_PO_4_, 1.0 g KNO_3_, 0.5 g MgSO_4_·7H_2_O, 0.5 g KCl, 0.2 g glucose, 0.2 g sucrose, 15.0 g agar, 1 L deionized water), ISP3 medium and pine needle medium. ISP3 medium was prepared as follows: 20 g of oat meal was boiled and, after cooling to below 55 °C, filtered with thin gauze. The filtrates were autoclaved. Then, 1 mL of sterile trace salt solution (0.1% FeSO_4_·7H_2_O, 0.1% MnCl_2_·4H_2_O, and 0.1% ZnSO_4_·7H_2_O) was supplemented per litre of medium. Pine needle medium was prepared by homogenizing 20 g of *Pinus massoniana* needles in a small volume of water, filtering the mixture, and bringing the filtrate to a final volume of 1000 mL. Then, 20 g of agar was added, and the medium was sterilised by autoclaving.

### ﻿Morphological studies

Mycelial blocks (approximately 5 mm in diameter) were placed at the centre of different media, including MEA, SNA, and OA (30 g of oatmeal was boiled and, after cooling to below 55 °C, filtered with thin gauze. The resulting filtrate was combined with 20 g of agar and sterilized by autoclaving.), and incubated in the dark at 25 °C, with sterilised pine needles added to stimulate sporulation. After 7 days of incubation, the colony characteristics were observed and recorded. Micromorphological structures were photographed under light microscopy, using a Canon 600D camera on a Nikon SMZ-1000 dissecting microscope (Tokyo, Japan), and an AxioCam MRc5 camera on a Zeiss Imager. M2 microscope (Carl Zeiss, Jena, Germany). Slide mounts were prepared using distilled water, lactic acid, and/or lacto-glycerol as mounting media. Morphological observations of reproductive structures were carried out using a Nikon SMZ-1000 dissecting microscope and a Zeiss Imager. M2 compound microscope equipped with differential interference contrast (DIC). For each morphological structure, thirty measurements were taken. Colour descriptions were determined according to Kornerup & Wanscher (Kornerup 1978).

### ﻿Molecular analyses

DNA was extracted by scraping fresh fungal mycelia off pure cultures and transferring them into 50 µL of extraction buffer (20 mM Tris [pH 8.0], 25 mM NaCl, 2.5 mM EDTA, 0.05% SDS) (Crous et al. 2019a). The supernatant was collected and used as the template for polymerase chain reaction (PCR) amplification.

The rDNA region of internal transcribed spacer (ITS), was amplified using the primer pair of ITS1 and ITS4 (White et al. 1990). 28S nrRNA gene (nLSU) was amplified using the primer pair of LR0R (Rehner and Samuels 1994) and LR5 (Vilgalys and Hester 1990). The RNA polymerase II (*RPB2*), translation elongation factor-1 α (*TEF1*), ß-tubulin (*TUB*), actin (*ACT*), and calmodulin (*CAL*) were amplified using the primers frpb2-5f/rpb2-7cr (Liu et al. 1999), 728F/2218R (Groenewald et al. 2013), T1/BT2B (O’Donnell and Cigelnik 1997; Woudenberg et al. 2009), ACT-512f/ACT2rd (Carbone and Kohn 1999; Liu et al. 1999), and CAL-235F/CAL2RD (Liu et al. 1999; Quaedvlieg et al. 2012). PCR amplification was carried out in a 25 µL reaction mixture consisting of 50–100 ng template DNA, 1.25 U Taq polymerase (Vazyme Biotech Co., Ltd., China), 200 μm dNTP, 0.5 μm of each primer, and 5% (v/v) dimethyl sulfoxide (DMSO). The PCR conditions were as follows: 95 °C for 3 min, followed by 30 cycles of 95 °C for 1 min, 50–55 °C for 1 min, and 72 °C for 1 min. The final extension step was 72 °C for 10 min. The amplified products were sequenced at Sangon Biotech (Nanjing, Jiangsu, China).

To determine the taxonomic placement of the isolates, sequences of *Dissoconiaceae* were first analysed to clarify their phylogenetic position. To further confirm their systematic status, additional phylogenetic analyses were conducted at both the family level (for the new genus) and the genus level (for the new species). To achieve broader taxonomic coverage, the selection of genetic regions for this study was based on previously established markers for each group. The following loci were used: ITS, nLSU, *RPB2* and TEF1 for *Dissoconiaceae* taxa (Crous et al. 2009c) and *Extremaceae* Quaedvl. & Crous taxa (Quaedvlieg et al. 2014), ITS, nLSU and *RPB2* for *Zasmidium* Fr. (Fries 1849) and *Mycosphaerellaceae* taxa (Crous et al. 2020b; Bakhshi et al. 2021; Rajeshkumar et al. 2021; Bakhshi and Braun 2022), ITS, nLSU, *RPB2*, *TUB* and EF for *Sphaerulina* Sacc. (Quaedvlieg et al. 2013), and ITS, nLSU, *RPB2*, *TEF1*, *ACT* and *CAL* for *Neocatenulostroma* (Punithalingam 1969). All of the sequences were deposited in NCBI GenBank (Suppl. material 1: table S1–S8). Each dataset was aligned with MAFFT v.74 (Katoh and Standley 2013) with the G-INS-I iterative refinement algorithm and manually adjusted in BioEdit v.7.0.5.3 (Hall 1999). The alignments of individual loci were subsequently concatenated using PhyloSuite v.1.2.2 (Zhang et al. 2020).

Maximum Likelihood (ML) and Bayesian Inference (BI) analyses were carried out by using RAxML v.8.2.10 (Stamatakis 2014) and MrBayes 3.2.6 (Ronquist et al. 2012), respectively. For ML analysis, nodal support was evaluated with 1000 rapid bootstrap replicates under default parameters. For BI, the optimal partitioning scheme and substitution models were selected with ModelFinder (Kalyaanamoorthy et al. 2017) via the “greedy” algorithm, with using the corrected Akaike information criterion (AICc). Four Markov chain Monte Carlo chains (one cold) were constructed for 5,000,000 generations, with sampling every 1000 generations. Convergence was confirmed when the average standard deviation of split frequencies fell below 0.01. The first 25% of trees were discarded as burn-in, and the remaining trees were used to construct a majority-rule consensus tree and estimate posterior probabilities (BPP).

Phylogenetic trees were visualized using FigTree version 1.4.4 (Rambaut 2018). Branches that received bootstrap supports for ML (≥ 75%) and BPP (≥ 0.95) were considered as significantly supported.

### ﻿Divergence time estimation

A time-calibrated phylogeny for the *Capnodiales* s. lat. was constructed based on a three-locus datasets (ITS + nLSU + *RPB2*) (Suppl. material 1: table S9). This dataset includes 136 species of *Capnodiales* s. lat., as well as reference taxa corresponding to fossil calibration. For evolutionary calibration, our calibration strategy was based on two well-established points that have been consistently applied across multiple publications, including Phukhamsakda et al. 2016; Pérez-Ortega et al. 2016; Liu et al. 2017; Zhang et al. 2019; Hongsanan et al. 2020. Specifically, the crown age of *Capnodiales* was constrained using a fossil from *Metacapnodiaceae*, with a normal distribution (mean = 100, SD = 150; 97.5% CI = 346 Mya). Additionally, the crown age of the *Dothideomycetes* was calibrated using the secondary calibration, with a normal distribution (mean = 290, SD = 30, and 97.5% of CI = 349 Mya).

Divergence times were estimated using BEAST v.2.6.5 (Bouckaert et al. 2014). An XML (Extensible Markup Language) file was generated with BEAUti (version 2). The rates of evolutionary changes at nuclear acids were estimated using ModelTest (version 3.7) with the GTR substitution model (Posada and Crandall 1998). Divergence time and corresponding CIs were conducted with a log-normal relaxed molecular clock and the Yule speciation priority. After 10,000,000 generations, the first 10% were removed as burn-in. The log file was checked for convergence with Tracer (version 1.54). A Maximum Clade Credibility (MCC) tree was then summarised with TreeAnnotator v.2.6.5, with nodes having posterior probability (PP) values above 0.8 being annotated.

## ﻿Results

### ﻿Phylogenetic analyses

During an investigation of endophytic fungi colonizing pine trees, we identified novel taxonomic entities within the order *Capnodiales* s. lat. These fungi exhibit distinct morphological characteristics that distinguish them from all previously known species. Based on integrated morphological observations and phylogenetic analyses, we establish that these taxa represent three novel genera and six novel species, which are distributed across six families: *Capnodiaceae*, *Cladosporiaceae*, *Mycosphaerellaceae*, *Dissoconiaceae*, *Extremaceae*, and *Teratosphaeriaceae*.

### ﻿Molecular phylogeny

#### ﻿*Capnodiales* s. lat. phylogeny based on combined ITS + nLSU + *RPB2* + *TEF1* sequence data (Fig. 1)

We used 136 sequences representing 131 species within *Capnodiales* s. lat. for phylogenetic analysis (Suppl. material 1: table S1), with *Elsinoe phaseoli* Jenkins as the outgroup (Hongsanan et al. 2020). The dataset has a total aligned length of 1847 characters, including 818 sites from ITS, 1029 from nLSU, 1079 sites from *RPB2*, and 963 from *TEF1*. ModelFinder suggested the GTR + F + I + G4 model for the Bayesian inference (BI), resulted in an average standard deviation of split frequencies of 0.066661. Both Bayesian inference (BI) and Maximum Likelihood (ML) analyses produced highly congruent topological structures at the generic level.

**Figure 1.**
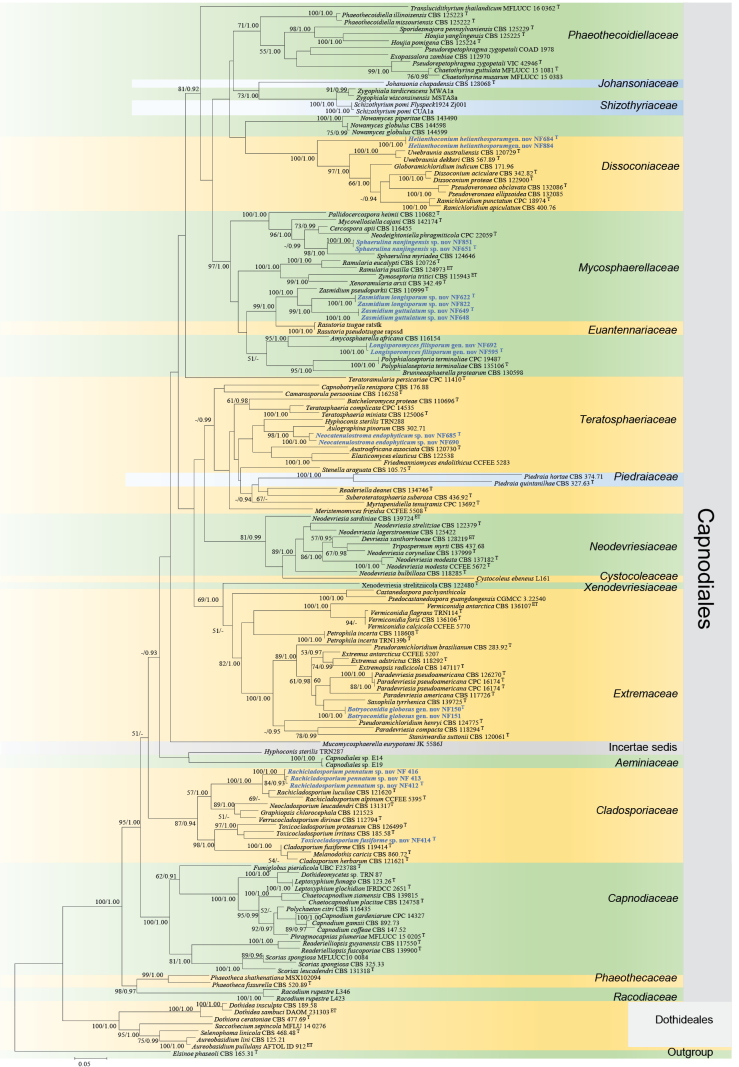
Phylogram of *Capnodiales* s. lat. based on a combined ITS + nLSU + *RPB2* + *TEF1* sequence dataset, inferred from maximum likelihood and Bayesian analyses. Numbers above the branches indicate ML bootstrap values (left, ≥ 50%) and Bayesian posterior probabilities (right, ≥ 0.90). The isolates from this study are marked in blue.

The phylogenetic results (Fig. 1) revealed that the newly obtained isolates are distributed across several families within *Capnodiales*, supporting the introduction of three novel genera and six novel species. The novel genus *Helianthoconium* (NF684, NF884) is placed in *Dissoconiaceae*; the novel genus *Botryoconidia* (NF150, NF151) is assigned to *Extremaceae*; the novel genus *Longisporomyces* (NF595, NF692), along with the novel species *Sphaerulina nanjingensis* (NF651, NF851), *Zasmidium longisporum* (NF622, NF822), and *Zasmidium guttulatum* (NF648, NF649), are placed within *Mycosphaerellaceae*; the novel species *Rachicladosporium pennatum* (NF412, NF413, NF416) and *Toxicocladosporium fusiforme* (NF414) belong to *Cladosporiaceae*; and *Neocatenulostroma endophyticum* (NF685, NF690) is placed in *Teratosphaeriaceae*. All the novel taxa form well-supported independent lineages in their respective clades.

#### ﻿*Dissoconiaceae* phylogeny based on combined ITS + nLSU sequence data (Fig. 2)

We used 23 sequences representing 22 species within *Dissoconiaceae* and 2 sequences from our specimen for phylogenetic analysis (Suppl. material 1: table S2), with *Myrmecridium schulzeri* (Sacc.) Arzanlou, W. Gams & Crous as the outgroup (Li et al. 2012). The dataset has a total aligned length of 1504 characters, including 551 sites from ITS and 953 from nLSU. ModelFinder suggested the GTR + F + I + G4 model for the Bayesian inference (BI), resulted in an average standard deviation of split frequencies of 0.003969. Both Bayesian inference (BI) and Maximum Likelihood (ML) analyses produced highly congruent topological structures at the generic level.

**Figure 2.**
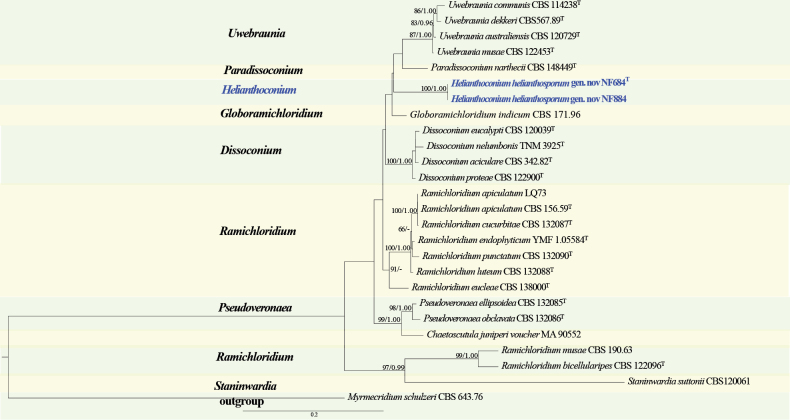
Phylogram of *Dissoconiaceae* based on a combined ITS + nLSU sequence dataset, inferred from maximum likelihood and Bayesian analyses. Numbers above the branches indicate ML bootstrap values (left, ≥ 50%) and Bayesian posterior probabilities (right, ≥ 0.90). The isolates from this study are marked in blue.

*Dissoconiaceae* formed a distinct clade that was clearly separated from other members of *Mycosphaerellales* (Crous et al. 2020b). The phylogenetic tree (Fig. 2) comprising the core genera of *Dissoconiaceae* —*Dissoconium* de Hoog, Oorschot & Hijwegen, *Globoramichloridium* Y. Marín & Crous, *Paradissoconium *, *Pseudoveronaea* Crous & Batzer, *Ramichloridium* Stahel ex de Hoog, *Staninwardia* B. Sutton, and *Uwebraunia* Crous & M.J. Wingf. —yielded highly congruent topologies between Bayesian inference (BI) and Maximum Likelihood (ML) analyses at the generic level. These results are consistent with previously published studies (Crous et al. 2019a), reinforcing the robustness of the generic relationships within *Dissoconiaceae*. Our phylogenetic reconstruction of *Dissoconiaceae* (Fig. 2) revealed ten distinct lineages, corresponding to eight previously recognized genera, one outgroup, and a unique, unclassified lineage. The new genus forms a distinct lineage within the family, clustering as a well-supported clade that is closely related to but clearly separable from other recognized genera. Therefore, we introduce a novel monotypic genus, *Helianthoconium*, within the family *Dissoconiaceae*.

#### ﻿*Extremaceae* phylogeny based on combined ITS + nLSU sequence data (Fig. 3)

We used 16 species representing sequences within *Extremaceae* and 2 sequences from our specimens for phylogenetic analysis (Suppl. material 1: table S3), with *Dothiora ceratoniae* (Quaedvl., Verkley & Crous) as the outgroup. The dataset has a total aligned length of 1355 characters, including 539 sites from ITS and 816 from nLSU. ModelFinder suggested the GTR + F + I + G4 model for the Bayesian inference (BI), resulted in an average standard deviation of split frequencies of 0.003886. Both Bayesian inference (BI) and Maximum Likelihood (ML) analyses produced highly congruent topological structures at the generic level.

**Figure 3.**
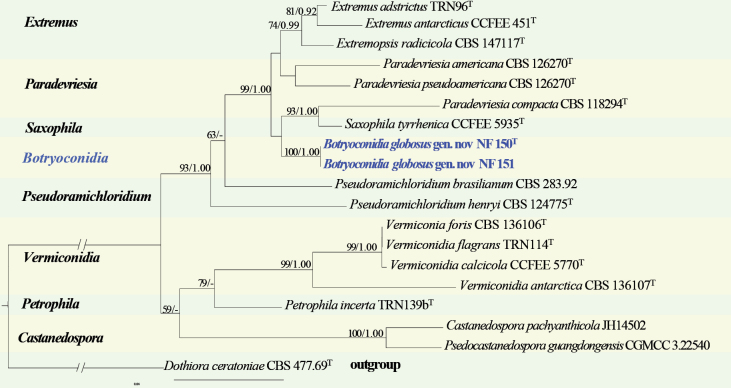
Phylogram of *Extremaceae* based on a combined ITS + nLSU sequence dataset, inferred from maximum likelihood and Bayesian analyses. Numbers above the branches indicate ML bootstrap values (left, ≥ 50%) and Bayesian posterior probabilities (right, ≥ 0.90). The isolates from this study are marked in blue.

**Figure 4.**
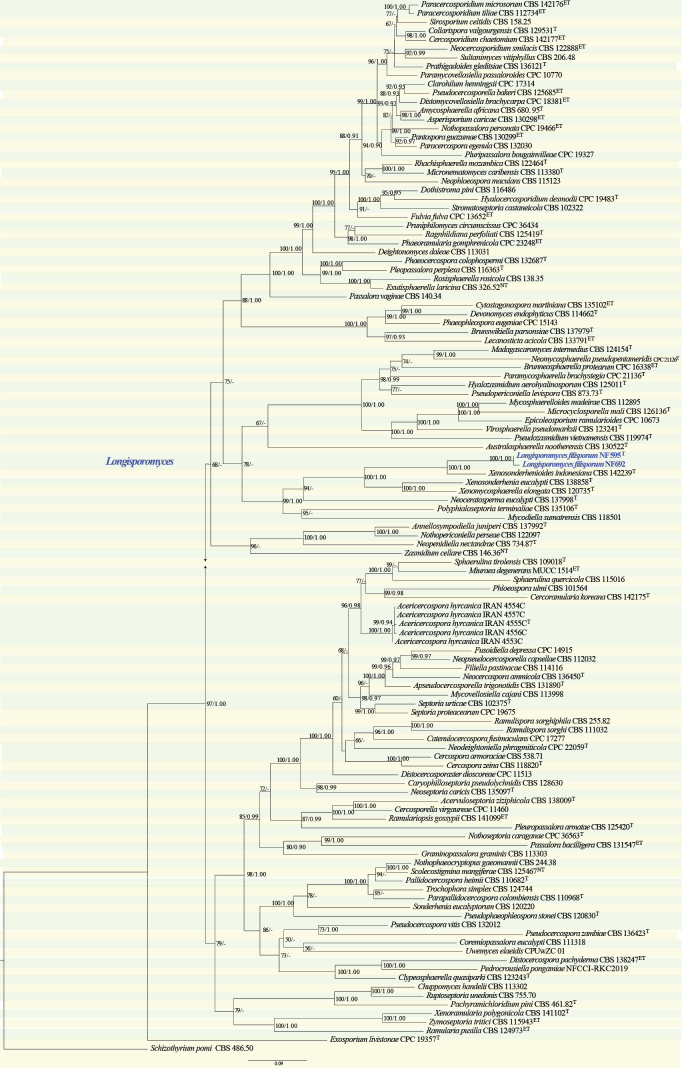
Phylogram of *Mycosphaerellaceae* based on a combined ITS + nLSU + *RPB2* sequence dataset, inferred from maximum likelihood and Bayesian analyses. Numbers above the branches indicate ML bootstrap values (left, ≥ 50%) and Bayesian posterior probabilities (right, ≥ 0.90). The isolates from this study are marked in blue.

*Extremaceae* and *Paradevriesiaceae* are considered taxonomic synonyms, representing a single distinct lineage within *Mycosphaerellales* (Crous 2009; Hongsanan et al. 2020). Our phylogenetic analyses of *Extremaceae* encompass all eight genera currently classified under this family, viz. *Castanedospora* G. Delgado & A.N. Mill., *Extremus* Quaedvl. & Crous, *Paradevriesia* Crous, *Petrophila* de Hoog & Quaedvl, *Pseudocastanedospora*, *Pseudoramichloridium* Cheew. & Crous, *Saxophila* Selbmann & de Hoog, and *Vermiconidia* Egidi & Onofri. Notably, five of them-- *Castanedospora*, *Extremopsis*, *Petrophila*, *Pseudocastanedospora*, and *Saxophila*--are recently established monotypic genera. Since the strains NF 150 and NF 151 do not nest within any existing genus and represent a well-separated lineage (100/1.00) within *Extremaceae*, we propose *Botryoconidia* as a novel genus, relating to *Saxophila* and *Paradevriesia*, (Fig. 4).

#### ﻿*Mycosphaerellaceae* phylogeny based on combined ITS + nLSU + *RPB2* sequence data (Fig. 4)

We used 117 sequences representing 113 species within *Dissoconiaceae* and 2 sequences from our specimen for phylogenetic analysis (Suppl. material 1: table S4), with *Schizothyrium pomi* (Mont. & Fr.) as the outgroup (Bakhshi and Braun 2022). The dataset has a total aligned length of 2173 characters, including 629 sites from ITS, 741 for nLSU and 803 from *RPB2*. ModelFinder suggested the GTR + F + I + G4 model for the Bayesian inference (BI), resulted in an average standard deviation of split frequencies of 0.021911. Both Bayesian inference (BI) and Maximum Likelihood (ML) analyses produced highly congruent topological structures at the generic level.

Our phylogenetic analysis of *Mycosphaerellaceae* includes 108 genera currently classified within this family. The overall topology at the generic level is consistent with those shown in previous studies (Videira et al. 2017; Bakhshi et al. 2021; Rajeshkumar et al. 2021; Bakhshi and Braun 2022). Two strains (NF595 and NF692, Fig. 4) isolated from endophytic fungi associated with *Pinus densiflora* formed a highly supported clade distinct from all other genera in the *Mycosphaerellaceae*. Therefore, a novel monotypic genus, *Longisporomyces*, is introduced in the family *Mycosphaerellaceae*.

#### ﻿*Cladosporiaceae* phylogeny based on combined ITS + nLSU + *RPB2* sequence data (Fig. 5)

We used 43 sequences representing 38 species within *Cladosporiaceae* and 4 sequences from our specimen for phylogenetic analysis (Suppl. material 1: table S5), with *Extremus antarcticus* Quaedvl. & Crous as the outgroup. The dataset has a total aligned length of 2668 characters, including 826 sites from ITS, 866 for nLSU, and 976 for *RPB2*. ModelFinder suggested the GTR + F + I + G4 model for the Bayesian inference (BI), resulted in an average standard deviation of split frequencies of 0.003634. Both Bayesian inference (BI) and Maximum Likelihood (ML) analyses produced highly congruent topological structures at the generic level.

**Figure 5.**
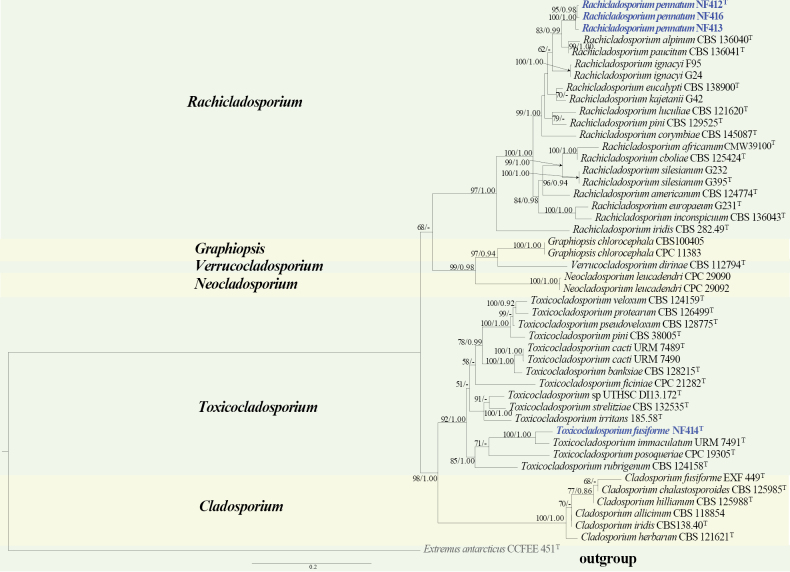
Phylogram of *Cladosporiaceae* based on a combined ITS + nLSU + *RPB2* sequence dataset, inferred from maximum likelihood and Bayesian analyses. Numbers above the branches indicate ML bootstrap values (left, ≥ 50%) and Bayesian posterior probabilities (right, ≥ 0.90). The isolates from this study are marked in blue.

Our phylogenetic analyses of *Cladosporiaceae* (Fig. 5), based on the combined ITS + nLSU + *RPB2* dataset, agree with the previous study (Bezerra et al. 2017) and support the proposal of two novel species, *Rachicladosporium pennatum* and *Toxicocladosporium fusiforme*. These two species cluster within the *Rachicladosporium* Crous, U. Braun & C.F. Hill and *Toxicocladosporium* Crous & U. Braun clade, respectively. Moreover, three strains of *Rachicladosporium pennatum* clustered as a robust lineage (BS = 100%, and BPP = 1.00), closely related to *R. alpinum* Egidi & Zucconi and *R. paucitum* Isola & Egidi, together forming an independent clade with a strong support (BS = 83%, and BPP = 0.99). *Toxicocladosporium fusiforme* forms a well-supported clade within the *Toxicocladosporium* clade, and groups with *T. immaculatum* J.D.P. Bezerra, Souza-Motta & Crous, formed an independent lineage with robust support (BS = 100%, and BPP = 1.00).

#### ﻿*Sphaerulina* phylogeny based on combined ITS + nLSU + *RPB2* + *TEF1* +*TUB* sequence data (Fig. 6)

We used 34 species representing sequences within *Sphaerulina* and 2 sequences from our specimens for phylogenetic analysis (Suppl. material 1: table S6), with *Pallidocercospora heimii* Crous as the outgroup. The dataset has a total aligned length of 2549 characters, including 600 sites from ITS, 805 of nLSU, 456 of *RPB2*, and 339 of *TEF1*, 349 of *TUB*. ModelFinder suggested the GTR + F + I + G4 model for the Bayesian inference (BI), resulted in an average standard deviation of split frequencies of 0.041653. Both Bayesian inference (BI) and Maximum Likelihood (ML) analyses produced highly congruent topological structures at the generic level.

**Figure 6.**
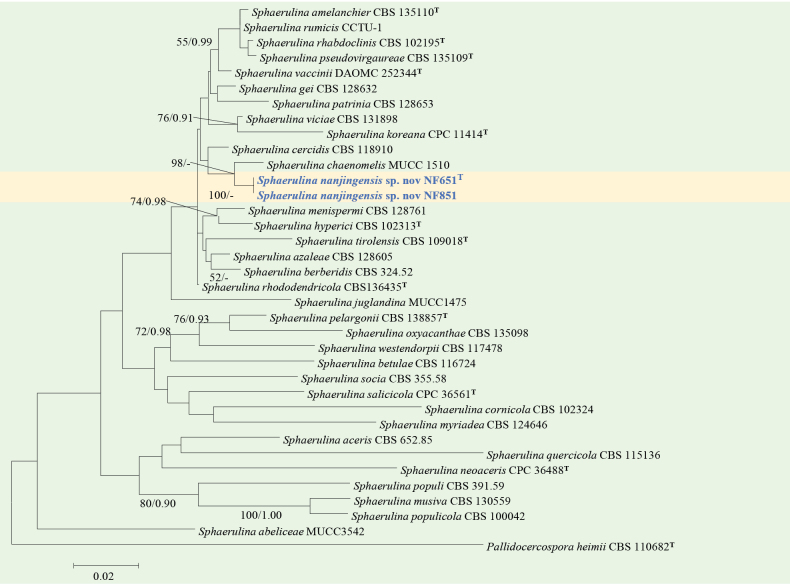
Phylogram of *Sphaerulina* based on a combined ITS + nLSU + *RPB2*+ *TEF1* + *TUB* sequence dataset, inferred from maximum likelihood and Bayesian analyses. Numbers above the branches indicate ML bootstrap values (left, ≥ 50%) and Bayesian posterior probabilities (right, ≥ 0.90). The isolates from this study are marked in blue.

Our phylogenetic analysis of *Sphaerulina* (Fig. 6), based on the combined ITS + nLSU + *RPB2* + *TEF1* + *TUB* dataset (Ali et al. 2021), revealed that two endophytic strains isolated from *Pinus densiflora*, formed a highly supported clade (BS = 100%), described here as the new species *Sphaerulina nanjingensis*.

#### ﻿*Teratosphaeriaceae* phylogeny based on combined ITS + nLSU + *RPB2* + *TEF1* + *ACT* + *CAL* sequence data (Fig. 7)

We used 102 sequences, representing 99 published species within *Teratosphaeriaceae* and two isolates obtained in this study, for phylogenetic analysis (Suppl. material 1: table S7), with *Staninwardia suttonii* Crous & Summerell as the outgroup. The dataset has a total aligned length of 3532 characters, including 650 sites from ITS, 891 of nLSU, 406 of *RPB2*, 553 of *TEF1*, 614 of *ACT* and 418 of *CAL*. ModelFinder suggested the GTR + F + I + G4 model for the Bayesian inference (BI), resulted in an average standard deviation of split frequencies of 0.09241. Both Bayesian inference (BI) and Maximum Likelihood (ML) analyses produced highly congruent topological structures at the generic level.

**Figure 7.**
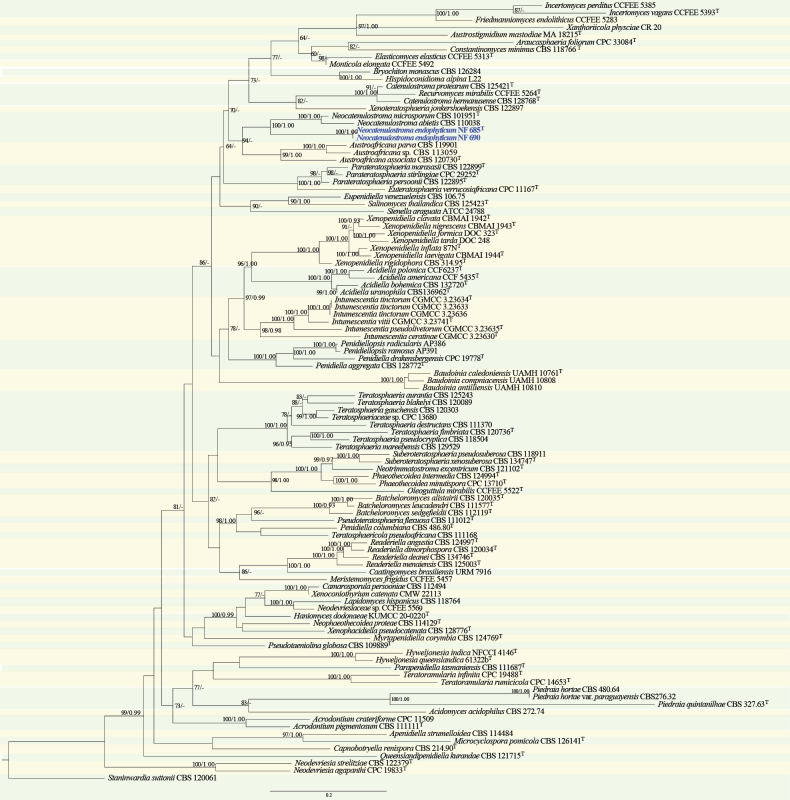
Phylogram of *Teratosphaeriaceae* inferred from maximum likelihood and Bayesian analyses based on a combined dataset (ITS + nLSU + *RPB2* + *TEF1* + *ACT* + *CAL*). Numbers above the branches indicate ML bootstrap values (left, ≥ 50%) and Bayesian posterior probabilities (right, ≥ 0.90). The isolates from the present study are marked in blue.

Our phylogenetic analyses of *Teratosphaeriaceae* (Fig. 7), based on the combined ITS + nLSU + *RPB2* + *TEF1* + *ACT* + *CAL* dataset, showed that two endophytic fungal strains isolated from *Pinus densiflora* in this study formed a distinct lineage with maximum support (BS = 100%, BPP = 1.00), described here as the new species *Neocatenulostroma endophyticum*. This species stably clusters within the *Neocatenulostroma* clade of *Teratosphaeriaceae*.

#### ﻿*Zasmidium* phylogeny based on combined ITS + nLSU + *RPB2* sequence data (Fig. 8)

We used 61 sequences, representing 60 published species within *Zasmidium* and four isolates obtained in this study, for phylogenetic analysis (Suppl. material 1: table S8), with *Nothopericoniella perseae-macranthae* (Hosag. & U. Braun) Videira & Crous as the outgroup (An et al. 2021). The dataset has a total aligned length of 2225 characters, including 605 sites from ITS, 780 of nLSU, and 840 of *RPB2*. ModelFinder suggested the GTR + F + I + G4 model for the Bayesian inference (BI), resulted in an average standard deviation of split frequencies of 0.006893. Both Bayesian inference (BI) and Maximum Likelihood (ML) analyses produced highly congruent topological structures at the generic level.

**Figure 8.**
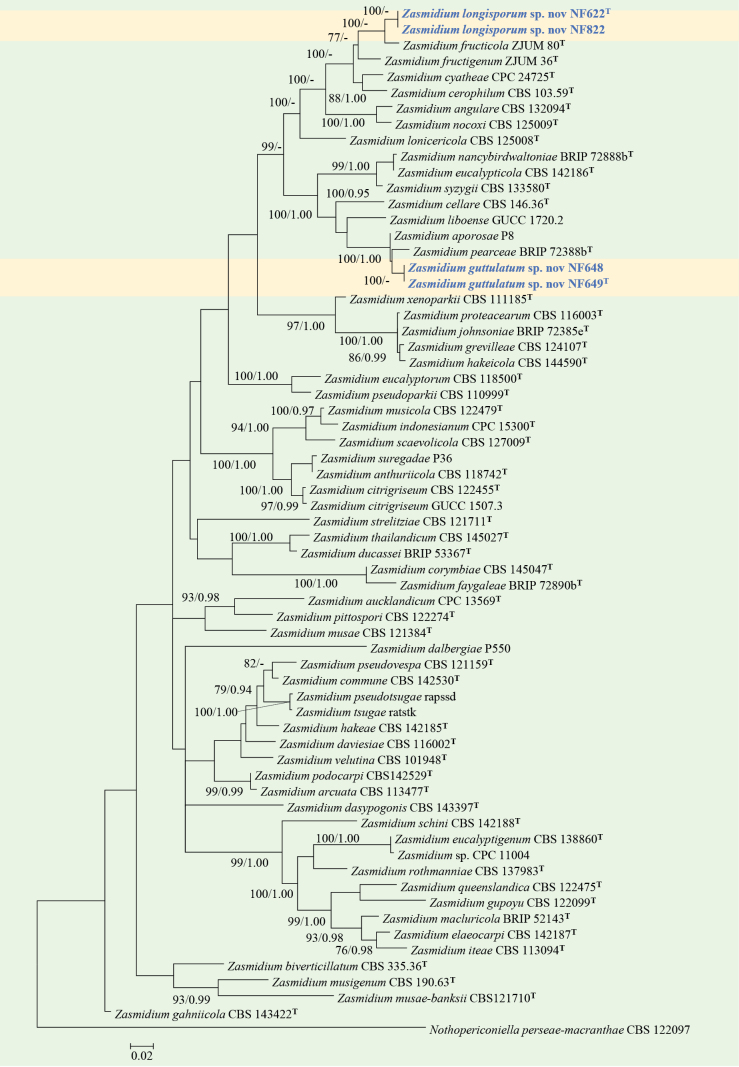
Phylogram of *Zasmidium* inferred from maximum likelihood and Bayesian analyses based on a combined dataset (ITS + nLSU + *RPB2*). Numbers above the branches indicate ML bootstrap values (left, ≥ 50%) and Bayesian posterior probabilities (right, ≥ 0.90). The isolates from the present study are marked in blue.

Our phylogenetic analyses of *Zasmidium* (Fig. 8), based on the combined ITS + nLSU + *RPB2* dataset, revealed that four endophytic fungal strains isolated from *Pinus densiflora* formed a well-supported clade (BS = 100), representing two new species: *Zasmidium longisporum* and *Zasmidium guttulatum*.

#### ﻿Divergence time estimation for *Capnodiales* s. lat. (Fig. 9)

A combination of ITS + nLSU + *RPB2* sequences (Suppl. material 1: table S9) were used to estimate the divergence time of *Capnodiales* s. lat. Previously reported members of *Capnodiales* s. lat. are primarily leaf epiphytes associated with honeydew (produced by insects), saprobes or parasites. In this study, endophytic fungi isolated from pine trees were found to cluster within the *Capnodiales* s. lat. We constructed a Maximum Cladistic Consensus (MCC) tree to elucidate the evolutionary framework of Capnodialean fungi and clarify the phylogenetic placement of these endophytic fungi within the order. The MCC tree provides an estimated temporal framework for the evolution of these fungi. The distinct phylogenetic positions of the proposed new genera and species within this framework offer further support for their establishment.

**Figure 9.**
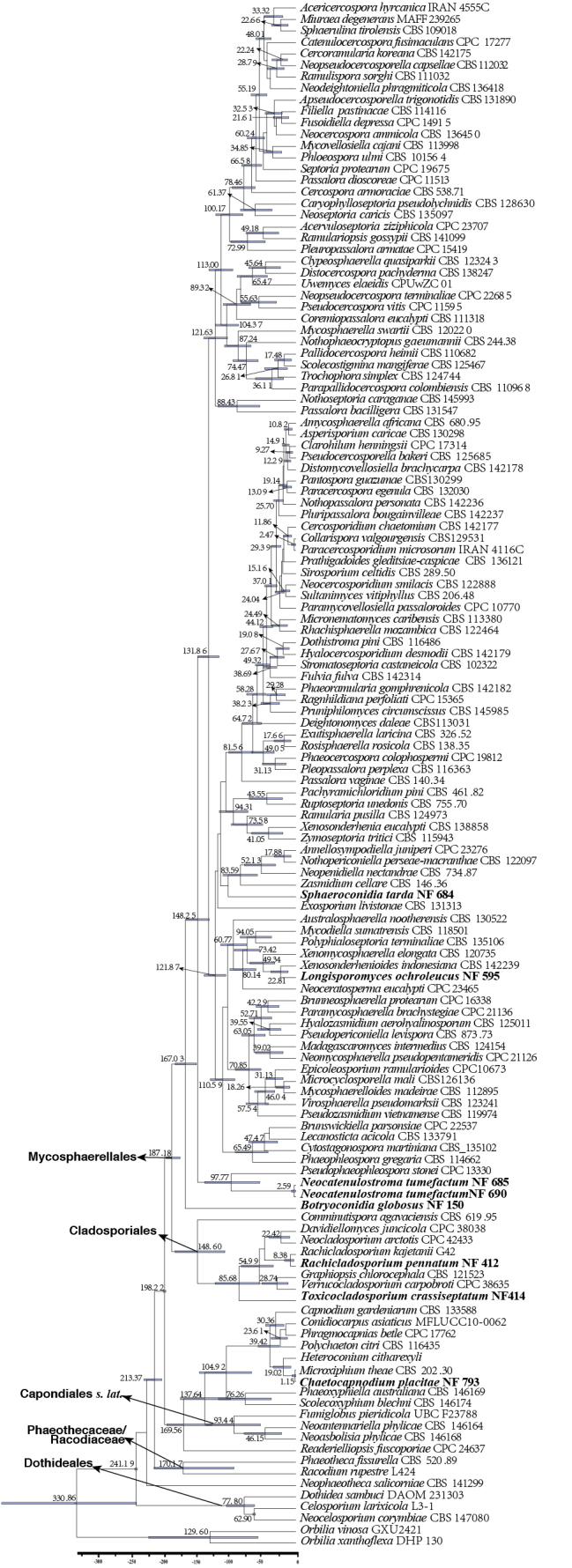
The maximum clade credibility (MCC) tree of families within *Capnodiales* s. lat. obtained from Bayesian approach (BEAST). Fossil calibration points and secondary calibrations used in this study are indicated with green dots. Bars represent 95% highest posterior density (HPD) intervals. The scale axis indicates divergence time in millions of years ago (Mya).

The MCC in Fig. 9 shows that the *Capnodiales* s. lat. emerged with a mean crown age of 213.37 Mya [95% highest posterior density (HPD): 196.28–200.18 Mya], consistent with the previous study (Hongsanan et al. 2020). Integrating morphological, ecological, and phylogenetic evidence, Abdollahzadeh et al. (Abdollahzadeh et al. 2020) subdivided *Capnodiales* s. lat. into seven orders: *Capnodiales* s. str., *Cladosporiales*, *Comminutisporales*, *Mycosphaerellales*, *Neophaeothecales*, *Phaeothecales*, and *Racodiales*. Our MCC tree further supports this taxonomic framework, indicating that these orders diverged during well-resolved temporal intervals. For example, *Mycosphaerellales*, the most species-rich order within this clade, diverged at a mean crown age of 187.18 Mya [95% HPD: 149.85–182.27 Mya]. Our analysis reveals that the endophytic fungi of pine trees, including *Helianthoconium helianthosporum*, *Xenosonderhenioides longisporomyces*, *Botryoconidia globosus*, cluster within the *Mycosphaerellales* lineage. Interestingly, our MCC tree indicates that *Botryoconidia globosus* appears as the earliest-diverging clade within the *Mycosphaerellales*. While this position could be influenced by taxon sampling, the overall structure of the MCC tree is nevertheless consistent with previous studies (Videira et al. 2017). This consistency underscores the unique phylogenetic and evolutionary status of *B. globosus*.

Our divergence time estimation also indicates that *Cladosporiales* and its sister order *Comminutisporales* diverged during the Late Jurassic, with a mean crown age of 148.60 Mya. This estimate provides an evolutionary context for the two-pine endophytic fungal strains NF412 and NF414, which represent *Rachicladosporium* and *Toxicocladosporium* respectively. Notably, *Toxicocladosporium*, representing the earliest-diverging clade within *Cladosporiales*, diverged with a mean stem age of 85.68 Mya [95% HPD: 54.91–121.98 Mya]. This divergence pattern suggests that *Toxicocladosporium* may occupy a basal phylogenetic position, potentially reflecting that is noteworthy for understanding the early evolution of Capnodialean fungi.

### ﻿Taxonomy

Our phylogenetic analyses revealed that the endophytic fungi from *Pinus densiflora*, *P. thunbergii*, and *P. elliottii*, present three novel genera and nine novel species in *Capnodiales* s. lat. Under the classification system of *Capnodiales* by Hongsanan et al. (2020), these genera are distributed in five families: *Cladosporiaceae*, *Mycosphaerellaceae*, *Dissoconiaceae*, *Extremaceae*, and *Teratosphaeriaceae*. The nine novel species are proposed based on both phylogenetic analyses and morphological characteristics, with the latter described below.

#### ﻿*Dissoconiaceae*

##### ﻿ Helianthoconium

Taxon classificationAnimaliaCapnodialesDissoconiaceae

X. Yu. Zhang, Q.Y. Zhang & B. Fan gen. nov.

21905525-ACD1-5D71-BDC3-39974CDDEB2C

C860146

###### Etymology.

Derived from “Helianthus” (sunflower) and conidium, referring to the sunflower seed-shaped conidia.

###### Type species.

*Helianthoconium helianthosporum* X. Yu. Zhang, Q.Y. Zhang & B. Fan, sp. nov.

###### Description.

Colonies are fluffy, fuzzy, olive to off-white, with intact or finely serrated margins and irregularly divided patterns. The hyphae are translucent to off-white, slender, branched, erect or curved, and smooth to slightly warty. Asexual state: Conidiophores erect, straight or flexuose, unbranched, multi-septate, smooth or slightly warty, medium-brown to brown. Conidiogenous cells terminal, pale brown to brown, smooth, subcylindrical, aseptate, somewhat darkened, unthickened scar. Conidia solitary, sunflower-seed-shaped, slightly tapering at the apex, aseptate. Sexual state not observed.

###### Notes.

Phylogenetically, *Helianthoconium* forms a separate clade within the family *Dissoconiaceae*, although internal relationships among genera are not fully resolved. Morphologically, *Helianthoconium* exhibits macroscopic characteristics similar to those of *Paradissoconium* and *Globoramichloridium*. However, it differs in both conidial and conidiophore morphology: *Paradissoconium* produces olive-shaped conidia on simple conidiophores, while *Globoramichloridium* forms elongated conidia on branched conidiophores. In contrast, *Helianthoconium* is characterised by aseptate, sunflower seed-shaped conidia and thick, elongated conidiophores. (Arzanlou et al. 2007; Marin-Felix et al. 2019; Crous et al. 2023).

##### ﻿ Helianthoconium helianthosporum

Taxon classificationAnimaliaCapnodialesDissoconiaceae

X. Yu. Zhang, Q.Y. Zhang & B. Fan sp. nov.

FB80F3B0-2E2D-598F-8BAF-EE5E19C029BF

C860147

Fig. 10

###### Etymology.

Derived from the Latin word “ helianthus,” indicating that it is named for distinctive morphology of its conidia, which closely resemble sunflower seeds.

###### Type.

**CHINA** • Jiangsu Province, Nanjing, Nanjing Forestry University, Baima Campus, fungal endophyte from *Pinus densiflora*, May 2023, Ben Fan and Xiuyu Zhang, NF684 (**holotype HMAS 352957**, culture ex-type CGMCC 3.28953).

###### Description.

Mycelium composed of hyaline, smooth or warty, aseptate, flexuous, branched, slender, 1.2–2.8 μm diam. Asexual state: Conidiophores erect, solitary, arising from hyphae, 2–15-septate, straight to flexuous, subcylindrical, brown, smooth, stout, unbranched, (57.4–) 63.3–122.6 (–161.6) × (2.3–) 3.1–4.7 (–4.7) μm. Conidiogenous cells terminal, pale brown to brown, smooth, subcylindrical, terminal, connected in series, (4.2–) 5.4–10.3 (–11.9) × (2.7–) 3.0–3.9 (–4.1) μm. Conidia terminal and lateral, aseptate, smooth, brown, sunflower seed-shaped, base truncate, (6.0–) 6.9–8.2 (–8.5) × (3–) 3.5–4 (–5) μm. Sexual state unknown.

###### Culture characteristics.

**Figure 10.**
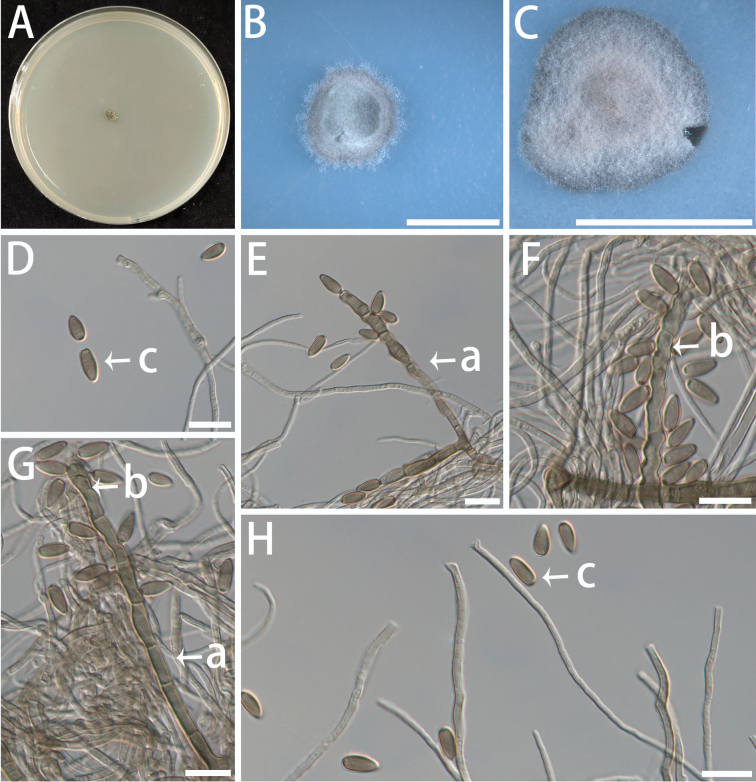
Morphological features of *Helianthoconium helianthosporum* (isolate NF684). A–C Colonies on MEA (A), SNA (B) and OA (C) at 25 °C for 10 days. A shown on a 90 mm Petri dish. D–H Conidiophores (a), conidiogenous cells (b), and conidia (c). Scale bars: 5 mm (B, C); 10 μm (D–H).

Colonies on MEA slow-growing, velvety to hairy, with entire margin; surface dark olivaceous-grey. On SNA and OA, the colonies appear off-white (Fig. 10A–C). The optimal temperature for growth was 20–25 °C, reaching 4–5 mm diam after 10 days on MEA. No growth was observed at 5 °C and 35 °C.

###### Additional specimen (paratype) examined.

**CHINA** • Jiangsu Province, Nanjing, Nanjing Forestry University, Baima Campus, fungal endophyte from *Pinus densiflora*, May 2023, Ben Fan and Xiuyu Zhang, paratype NF884.

###### Notes.

In the phylogenetic analyses of *Capnodiales* (Fig. 1) and *Dissoconiaceae* (Fig. 2), the isolate of *Helianthoconium helianthosporum* formed a well-supported clade within *Dissoconiaceae* (ML/BI = 100/1.00). BLAST analysis of the ITS sequence of NF684 against NCBI reveals a 10.78% (51/473 bp) difference from its closest known relative, indicating a clear genetic distinction. Although consistently placed within *Dissoconiaceae*, *Helianthoconium helianthosporum* differs from other genera in this family by its sunflower seed-shaped conidia (vs. broadly ellip soidal to globose in *Globoramichloridium* and cylindrical in *Paradissoconium*) and shorter conidiogenous cells (4.2–11.9 μm vs. 15–45 μm in *Paradissoconium narthecii*). Based on these phylogenetic and well-defined morphological differences, we propose the new genus *Helianthoconium*, with *H. helianthosporum* as the type species.

#### ﻿*Extremaceae*

##### ﻿ Botryoconidia

Taxon classificationAnimaliaCapnodialesExtremaceae

X. Yu. Zhang, Q.Y. Zhang & B. Fan gen. nov.

7F4827C8-BBCA-5E06-8430-E2B52A89B360

C860148

###### Etymology.

Derived from the Latin words “ botrys” and “ conidium”, referring to the grape-like clustered arrangement of conidia.

###### Type species.

*Botryoconidia globosus* X. Yu. Zhang, Q.Y. Zhang & B. Fan, sp. nov.

###### Description.

Colonies olive, tomentose, with irregularly margins. Hyphae hyaline to pale brown, smooth to verruculose, septate, branched, varying in shape from straight to wavy or curved. Asexual state: Conidiophores reduced to conidiogenous cells or occasionally absent. Conidiogenous cells integrated, terminal or intercalary, hyaline to pale brown, smooth-walled, arising from vegetative hyphae, producing conidia either synchronously or successively. The apex of the conidiogenous cells often slightly swollen or constricted after conidium secession. Conidia developing directly on hyphae in an apical or lateral manner or on conidiophores, suboval to elliptical, smooth, occasionally aggregated in clusters. Sexual state not observed.

###### Notes.

*Extremaceae* was introduced by Quaedvlieg et al. (2014) and has since been expanded to include ten genera, reflecting growing research interest in this family. Most of the species in *Extremaceae* are rock-inhabiting taxa, saprobes, or isolated from soil. In this study, however, the genus *Botryoconidia*, represented by an endophytic strain from *Pinus densiflora*, forms a distinct lineage within *Extremaceae* (Fig. 4). Morphologically, *Botryoconidia* differs significantly from other genera in this family. While conidia in other *Extremaceae* genera are typically solitary or arranged in chains (Videira et al. 2017), those of *Botryoconidia* are oval-granular and aggregated in grape-like clusters.

##### ﻿ Botryoconidia globosus

Taxon classificationAnimaliaCapnodialesExtremaceae

X. Yu. Zhang, Q.Y. Zhang & B. Fan sp. nov.

4C2767D8-5E56-5BEA-A6F1-C975554B3745

C860149

Fig. 11

###### Etymology.

Derived from the Latin word “globosus” indicating that the Conidia is spherical.

**Figure 11.**
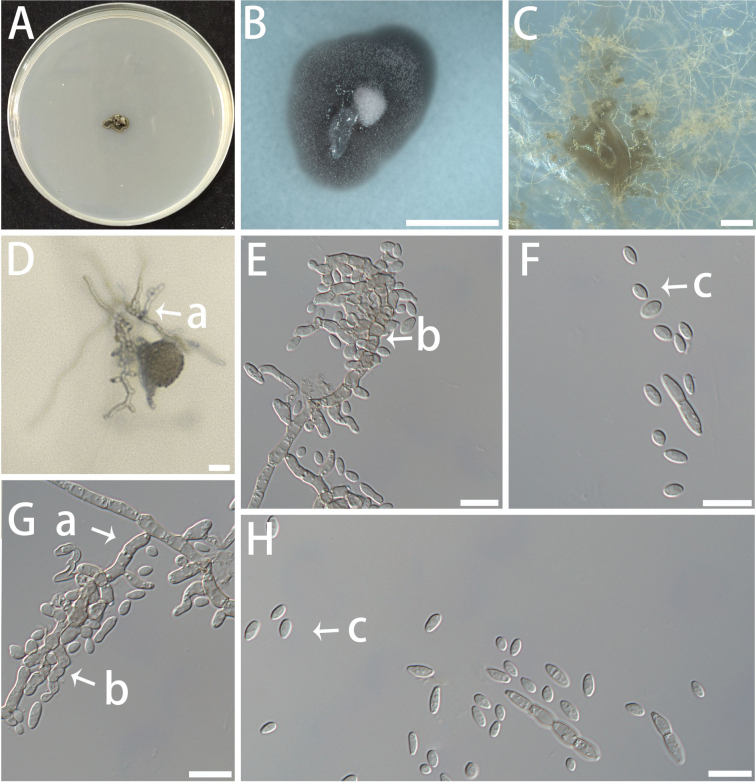
Morphology of asexual structures of *Botryoconidia globosus* (isolate NF150). A–C Colonies on MEA (A), OA (B) and SNA(C) at 25 °C for 10 days. A shown on a 90 mm Petri dish. D–H Conidiophores (a), conidiogenous cells (b), and conidia (c). Scale bars: 5 mm (B); 100 μm (C);10 μm (D–H).

###### Type.

**CHINA** • Jiangsu Province, Nanjing, Nanjing Forestry University, Baima Campus, fungal endophytes from *Pinus elliottii*, May 2023, Ben Fan and Xiuyu Zhang, NF150 (**holotype HMAS 352960**, culture ex-type CGMCC 3.28956).

###### Description.

Mycelium composed of hyaline to pale brown, smooth- to verruculose-walled, multi-septate, branched, slender, and uniform hyphae, 1.6–2.8 μm diam. Asexual state: Conidiophores reduced to conidiogenous cells or conidiogenous cells absent, (27.3–) 37.2–74.6 (–87.9) × (1.9–) 2.2–3.0 (–3.2) μm. Conidiogenous cells arising from the top of the hyphae or branching out from the sides of the hyphae, 0–1-septate, rough, short, and having few branches, (3.1–) 3.3–5.6 (–6.8) × (2.2–) 2.3–2.8 (–3.0) μm. Conidia developing directly from the end or side of the hyphae or from conidiogenous cells on conidiophores, oval-shaped to spindle-shaped, in clusters like grapes in bunches, translucent or hyaline, smooth, (2.3–) 2.7–5.0 (–6.2) × (1.5–) 1.6–2.4 (–3.0) μm. Sexual state unknown.

###### Culture characteristics.

Colonies on MEA and OA, with a compact surface, dark olive, and irregular edges. The optimal temperature for growth is 20–25 °C, reaching 5–8 mm in diameter in 10 days. No growth at 5 °C and 35 °C.

###### Additional specimen (paratype) examined.

**CHINA** • Jiangsu Province, Nanjing, Nanjing Forestry University, Baima Campus, fungal endophytes from *P. elliottii*, May 2023, Ben Fan and Xiuyu Zhang, paratype NF151.

###### Notes.

In the phylogenetic analyses of *Extremaceae* (Fig. 3), the isolate of *Botryoconidia globosus* clustered with *Paradevriesia* and *Saxophila* within the family, although the internal relationships among these genera were not well resolved. NF150 shows ITS sequence divergence of 9.98% (45/451 bp) from *Saxophila* (*S. tyrhenica*), supporting its phylogenetic distinctness. Morphologically, *B. globosus* has small (2.3–6.2 × 1.5–3.0 μm), hyaline, single-celled conidia from reduced conidiophores, while *S. tyrhenica* produces large (10.0–12.0 × 15.0–20.0 μm), multicellular, brown arthric conidia (Isola et al. 2016).

##### ﻿ Longisporomyces

Taxon classificationAnimaliaCapnodialesExtremaceae

X. Yu. Zhang, Q.Y. Zhang & B. Fan gen. nov.

E05B0A3E-73E0-5A5E-B76B-69DE11A8B8FF

C860150

###### Etymology.

Derived from the Latin words “longus” and “spora,” indicating that its spores are elongated.

###### Type species.

*Longisporomyces filisporum* X. Yu. Zhang, Q.Y. Zhang & B. Fan, sp. nov.

###### Description.

Colonies pale yellow to off-white fluffy, tomentose, with irregular edges. Hyphae verruculose or smooth, multi-septate, elongated, rarely branching, various in shapes, some are straight, some wavy or curved. Some hyphae aggregate into bundles and grow upward, forming thicker mycelial masses on MEA. Asexual state: Conidiophores and conidiogenous cells absent or conidiophores reduced to conidiogenous cells. Conidia are formed directly on the hyphae in an apical or lateral manner, slender filiform, straight or flexuous, smooth, multi-septate, and sometimes in chains. Sexual state not observed.

###### Notes.

The family *Mycosphaerellaceae* constitutes the most species-rich clade within *Capnodiales*, comprising approximately 1,000 described species with a global distribution and considerable ecological versatility (Bakhshi et al. 2015; Videira et al. 2017; Crous et al. 2020b; Rajeshkumar et al. 2021). Its members exhibit remarkable ecological plasticity, functioning as plant pathogens, endophytes, saprobes, and occasionally hyperparasites. They colonize a wide range of substrates or hosts, reflecting substantial adaptive diversity. Recent taxonomic revisions, aided by integrative morphogenomic approaches, have expanded the family to include more than 135 genera (Bakhshi et al. 2015; Videira et al. 2017; Crous et al. 2020b; Bakhshi et al. 2021; Rajeshkumar et al. 2021). In this study, we describe a new monotypic genus, *Longisporomyces*, within this family, typified by *Longisporomyces filisporum*, based on a combination of morphological and molecular characteristics.

##### ﻿ Longisporomyces filisporum

Taxon classificationAnimaliaCapnodialesExtremaceae

X. Yu. Zhang, Q.Y. Zhang & B. Fan sp. nov.

438BE076-D9C0-5D56-A6F4-CC6C6D6D7EAE

C860176

Fig. 12

###### Etymology.

referring to the distinctive filiform (thread-like) conidia produced by this species.

###### Type.

**CHINA** • Jiangsu Province, Nanjing, Nanjing Forestry University, Baima Campus, fungal endophytes from *Pinus densiflora*, May 2023, Ben Fan and Xiuyu Zhang, NF595 (**holotype HMAS 352956**, culture ex-type CGMCC 3.29108).

###### Description.

**Figure 12.**
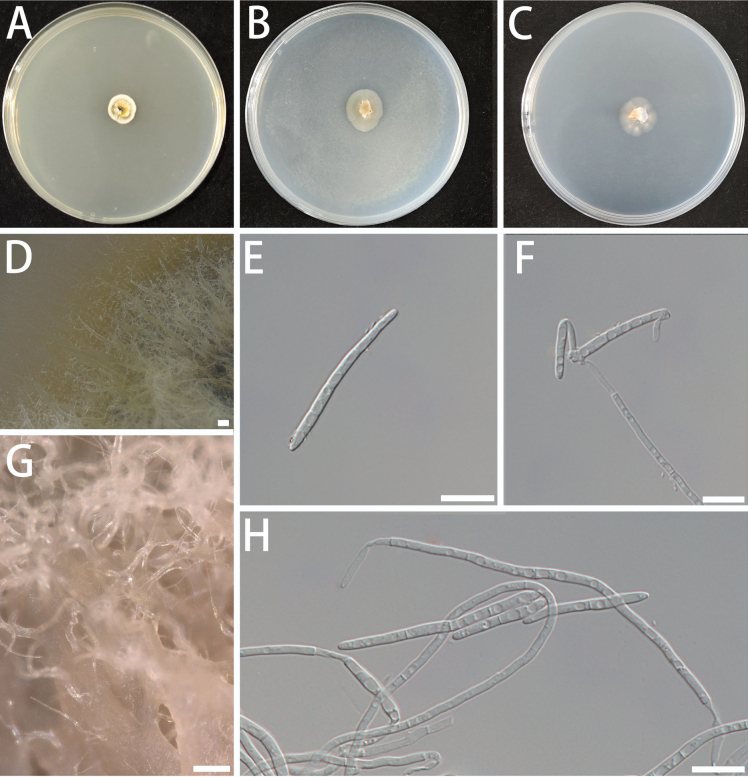
Morphological characteristics of *Longisporomyces filisporum* (isolate NF595). A–C Colonies on MEA (A), OA (B), and SNA (C) at 25 °C for 10 days on 90 mm Petri dishes. D, G Hyphal morphology on MEA. E, F, H Conidia. Scale bars: 100 μm (D, G); 10 μm (E, F, H)

Mycelium composed of hyaline, smooth, multi-septate, branched, uniform hyphae in width, slender, 1.4–2.6 μm diam. Many aerial hyphae are clustered in bundles, thicker at the base and tapering upwards on MEA (Fig. 12D, G). Asexual state: Conidiophores and conidiogenous cells absent or conidiophores reduced to conidiogenous cells. Conidiophores not obvious when present, and conidia developing directly from the ends or sides of hyphae. Conidia multiseptated, filiform or slightly flexuous, slender, hyaline, smooth, (9.9–) 14.3–35.3 (–52.2) × (1.5–) 2.0–3.0 (–3.5) μm (Fig. 12E, H). Sexual state unknown.

###### Culture characteristics.

Colonies on MEA, with a fluffy surface, pale yellow to off-white in colour, and irregular edges. In contrast, colonies grown on OA and SNA medium are transparent to white, dense, with irregularly lobed edges and few aerial hyphae. The optimal temperature for growth was 20–25 °C, reaching 8–12 mm diam in 10 days. No growth at 5 °C and 35 °C.

**Additional specimen (paratype) examined. CHINA** • Jiangsu Province, Nanjing, Nanjing Forestry University, Baima Campus, fungal endophytes from *Pinus densiflora*, May 2023, Ben Fan and Xiuyu Zhang, paratype NF692.

###### Notes.

In the present study, the novel genus *Longisporomyces* was established to accommodate isolates producing filiform conidia, represented by *Longisporomyces filisporum* (NF595 and NF692). Phylogenetic analyses within *Mycosphaerellaceae* (Fig. 4) revealed that *Longisporomyces* forms a well-supported clade (BS = 100%, BPP = 1.00) and is sister to the clade containing *Xenosonderhenioides indonesiana* C. Nakash., Videira & Crous. It is clearly distinct from the genera *Xenosonderhenia* Crous and *Xenomycosphaerella* Quaedvl. & Crous.

Phylogenetically, *Longisporomyces* forms a well-supported, distinct lineage that is sister to a highly supported clade containing *Xenomycosphaerella*, *Xenosonderhenia* and *Xenosonderhenioides*. While we retain *Longisporomyces* as a distinct genus due to its genomic divergence and distinct morphological characteristics. *L. filisporum* NF595 shows ITS sequence divergence of 3.98% (19/477 bp) from *Xenosonderhenioides indonesiana*, 7.78% (40/514 bp) from *Xenomycosphaerella elongata*, and 6.25% (30/480 bp) from *Xenosonderhenia eucalypti*. Morphologically, the generic type, *Longisporomyces filisporum*, is characterized by filiform and flexuous conidia, which are slightly narrower than those of *Xenosonderhenioides indonesiana* (1.5–3.5 μm vs. 5–6 μm) (Videira et al. 2017). In addition, conidia of *L. filisporum* are narrower than those of *X. elongata* (1.5–3.5 μm vs. 4–5 μm) and have more septa (0–3 vs. 1) (Crous et al. 2007c), and they are long-cylindrical, differing from the fusoid-ellipsoid conidia of *Xenosonderhenia eucalypti* (Crous et al. 2014b).

##### ﻿ Neocatenulostroma endophyticum

Taxon classificationAnimaliaCapnodialesExtremaceae

X. Yu. Zhang, Q.Y. Zhang & B. Fan sp. nov.

CD5A9533-31F1-5AEE-BCEC-87C8A7EFE840

C860177

Fig. 13

###### Type.

**CHINA** • Jiangsu Province, Nanjing, Nanjing Forestry University, Baima Campus, fungal endophyte from *Pinus densiflora*, May 2023, Ben Fan and Xiuyu Zhang, NF685 (**holotype HMAS 352961**, culture ex-type CGMCC 3. 28957).

###### Etymology.

Derived from the Latin word “endophyticum,” indicating its ecological niche as an endophyte.

###### Description.

**Figure 13.**
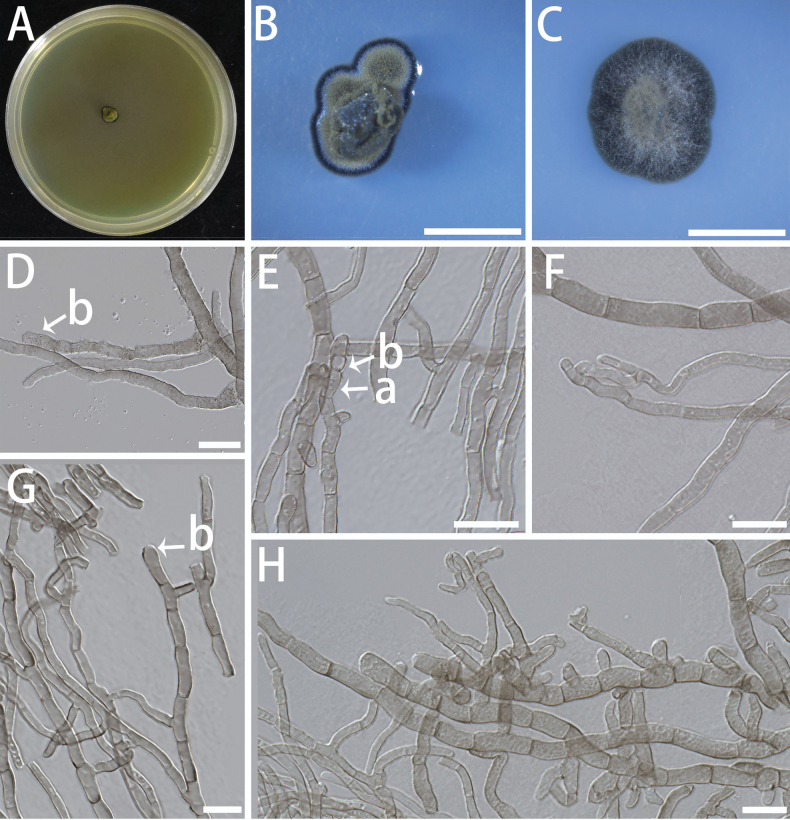
Morphology of *Neocatenulostroma endophyticum* (isolate NF685). A–C Colonies on MEA(A), OA (B) and SNA(C) at 25 °C for 10 days. A shown on a 90 mm Petri dish. D–H Conidiogenous cells (a) and conidia (b). Scale bars: 5 mm (B, C); 10 μm (D–H).

Mycelium composed of hyaline to pale brown, multi-septate, branched, smooth- to verruculose-walled hyphae, 2.5–3.9 μm. Asexual morph blastic-acropetal; conidiogenous cells developing from swollen hyphal segments, pale brown to brown, thick-walled, verruculose, straight to slightly curved, occasionally in short series Conidia produced successively at the apex of conidiogenous cells, forming acropetal chains; conidia hyaline to pale brown, verruculose, aseptate, ellipsoidal, relatively thick-walled, (5.2–) 6.0–10.4 (–13.5) × (2.6–) 3.5–4.5 (–4.9) μm (Fig. 13 D–H). Conidia frequently germinate laterally or apically by means of short, thin-walled germ tubes, which may elongate into hyaline hyphae or give rise to new conidial chains (Fig. 13 H). Although the conidia are relatively thick-walled and pigmented, they are not chlamydospores, as they are actively produced in conidial chains. Sexual state unknown.

###### Culture characteristics.

Colonies are black to dark olive in colour on MEA and OA, growing very slowly with a slightly fuzzy texture, dense in the centre and relatively loose at the edges. On SNA, aerial hyphae appear white and cottony. The optimal temperature for growth is 20–25 °C, reaching 3–6 mm diam in 10 d. No growth at 5 °C and 35 °C.

###### Additional material examined.

**CHINA** • Jiangsu Province, Nanjing, Nanjing Forestry University, Baima Campus, fungal endophytes from *Pinus densiflora*, May 2023, Ben Fan and Xiuyu Zhang, NF690.

###### Notes.

Two endophytic fungal isolates obtained from resistant *Pinus densiflora* formed a distinct lineage within the genus *Neocatenulostroma*, showing close phylogenetic affinity to *N. microsporum* (Joanne E. Taylor & Crous) Quaedvl. & Crous and *N. abietis* (Butin & Pehl) Quaedvl. & Crous. Morphologically, *Neocatenulostroma* species belong to a group of black yeast-like melanized Capnodialean microfungi (Markovskaja et al. 2016). However, unlike the present endophytic isolates, both *N. microsporum* and *N. abietis* are plant pathogens, reported from South Africa and Europe, respectively. The new taxon, *N. endophyticum*, differs from *N. microsporum* by 6.19% (28/452 bp) in the ITS region, and can be further distinguished by producing slightly shorter and more variably shaped conidia (5.2–13.5 × 2.6–4.9 μm) , although the size ranges partly overlap with those of its closest relatives, which are 13–15 × 5.5–6 μm in *N. microsporum* (Quaedvlieg et al., 2014) and 8–24 × 5–7 μm in *N. abietis* (Butin et al. 1996).

##### ﻿ Sphaerulina nanjingensis

Taxon classificationAnimaliaCapnodialesExtremaceae

X. Yu. Zhang, Q.Y. Zhang & B. Fan sp. nov.

6A795F7B-8E91-5AA4-8A96-2C5CCB4F0AD8

C860179

Fig. 14

###### Type.

**CHINA** • Jiangsu Province, Nanjing, Nanjing Forestry University, Baima Campus, fungal endophytes from *Pinus densiflora*, May 2023, Ben Fan and Xiuyu Zhang, NF651 (**holotype HMAS 352955**, culture ex-type CGMCC 3. 28952).

###### Etymology.

Derived from “Nanjing,” indicating that this species was first discovered in Nanjing.

###### Description.

**Figure 14.**
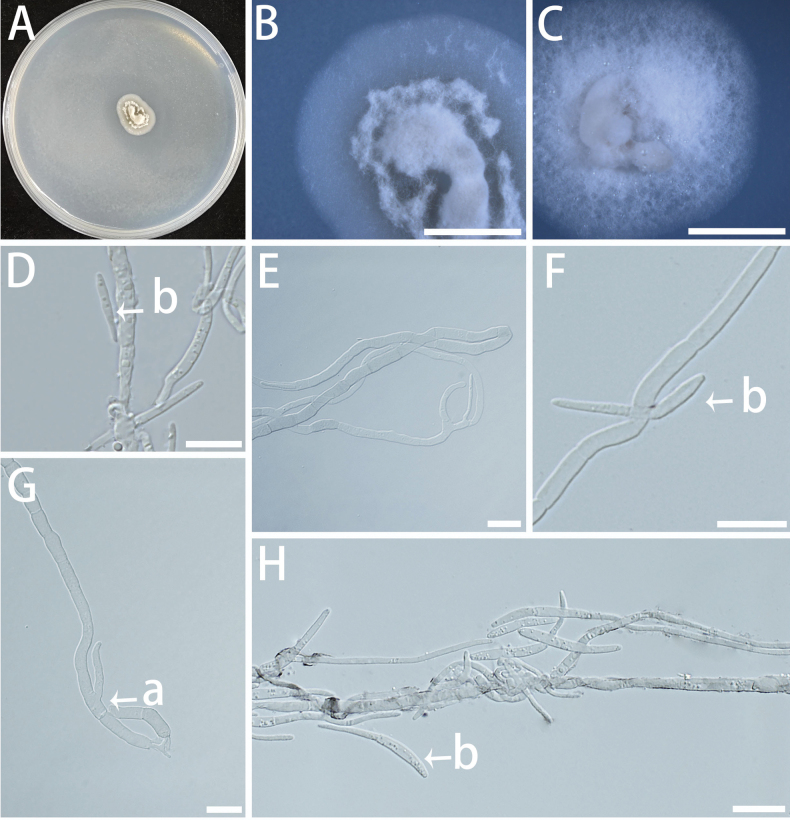
Morphology of *Sphaerulina nanjingensis* (isolate NF651). A–C Colonies on OA (A, B) and SNA (C) at 25 °C for 10 days. A shown on a 90 mm Petri dish. D–H Conidiogenous cells (a) and conidia (b). Scale bars: 5 mm (B, C); 10 μm (D–H).

Mycelium composed of hyaline, smooth to slightly rough-walled, multi-septate, branched and slender hyphae, 1.3–2.2 μm in diameter. Asexual state: Conidiophores reduced to conidiogenous cells. Conidiophores are not clearly differentiated; Conidiogenous cells are integrated, terminal or intercalary, hyaline, smooth-walled, monoblastic to polyblastic, producing conidia in acropetal chains through blastic conidiogenesis; conidia are produced directly from the apical or lateral sides of hyphae. Conidia multiseptated, long, filiform, falcate, slender, hyaline, smooth, (10.0–) 14.7–27.4 (–38.3) × (1.4–) 1.6–2.1 (–2.2) μm (Fig. 14D–H). Sexual state unknown.

###### Culture characteristics.

Colonies on SNA with a floccose to woolly surface, off-white to translucent in appearance, and with a regular, well-defined margin. On OA, aerial hyphae are concentrated in the middle area with very few around it. The optimal temperature is 20–25 °C, reaching 9–13 mm in diameter in 10 days on OA. No growth at 5 °C or 35 °C.

###### Additional material examined.

**CHINA** • Jiangsu Province, Nanjing, Nanjing Forestry University, Baima Campus, fungal endophytes from *Pinus densiflora*, May 2023, Ben Fan and Xiuyu Zhang, NF851.

###### Notes.

Two isolates, NF651 and NF851, identified as *S. nanjingensis*, are phylogenetically related to *S. chaenomelis*. Nevertheless, they can be clearly distinguished based on both morphological characteristics and multi-locus phylogeny. Morphologically, conidia of NF651 are significantly smaller, measuring (10.0–) 14.7–27.4 (–38.3) × (1.4–) 1.6–2.1 (–2.2) μm, whereas those of *S. chaenomelis* are considerably larger, (10–) 30–38 (–50) × (2–) 2.5–3 (–4) μm. Ecologically, NF651 was isolated as an endophyte from *Pinus densiflora*, while *S. chaenomelis* is known as a pathogen of *Chaenomeles sinensis* (Crous et al. 2013a). Although some endophytic species are known to display pathogenic behaviour under certain conditions, the distinct host associations and lifestyles of these two taxa suggest ecological divergence. At the molecular level, NF651 differs from *Sphaerulina chaenomelis* MUCC 1510 (type) by 35 nucleotide substitutions across 2315 aligned base pairs from the combined ITS, nLSU, *RPB2*, *TEF1* and *TUB* gene regions, corresponding to a divergence of 1.51%.The ITS and nLSU regions are relatively conserved among *Sphaerulina* species, whereas most variations occur in the protein-coding genes—3.78% (13/344 bp) in *RPB2*, 2.22% (7/314 bp) in *TEF1*, and 3.71% (14/377 bp) in *TUB*—which exceed the typical intraspecific range (Jeewon and Hyde 2016) and thus support its recognition as a distinct species.

##### ﻿ Rachicladosporium pennatum

Taxon classificationAnimaliaCapnodialesExtremaceae

X. Yu. Zhang, Q.Y. Zhang & B. Fan sp. nov.

5E381E42-96AF-599D-A42D-E9A6726EE37F

C860180

Fig. 15

###### Type.

**CHINA** • Jiangsu Province, Nanjing, Nanjing Forestry University, Baima Campus, endophytes from *Pinus thunbergii*, May 2023, Ben Fan and Xiuyu Zhang, NF412 (**holotype HMAS 354160**, culture ex-type CGMCC 3. 28959).

###### Etymology.

Derived from the Latin word “pennatum”, the specific epithet refers to the distinctive pinnate arrangement of conidiophores and conidia.

###### Description.

**Figure 15.**
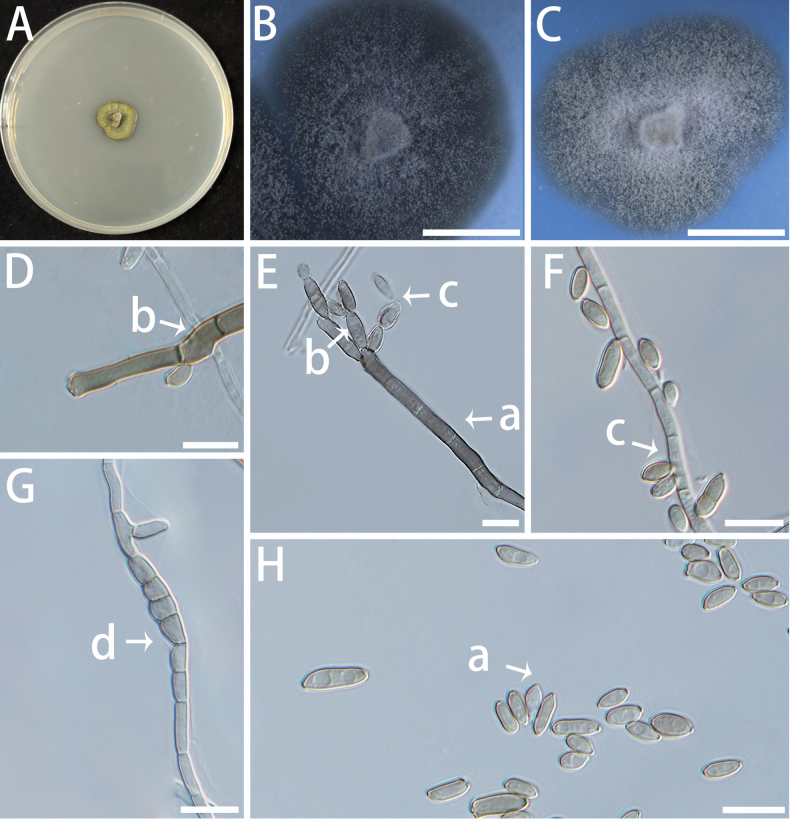
Morphology of *Rachicladosporium pennatum* (isolate NF412). A–C Colonies on MEA (A), OA (B) and SNA (C) at 25 °C for 10 days. A shown on a 90 mm Petri dish. D–H Conidiophores (a), conidiogenous cells (b), and conidia (c, d) arranged in chains. Letters “c” and “d” indicate two distinct conidial morphologies. Scale bars: 5 mm (B, C); 10 μm (D–H).

Mycelium composed of branched, multi-septate, subhyaline to pale brown hyphae, smooth-walled, 1.9–3.3 μm diam. Asexual state consisting of two conidiation modes: Blastic conidiation: Conidiophores arising laterally or terminally from hyphae, subcylindrical to clavate, multi-septate, pale brown, smooth, (41.9–) 57.6–150.0 (–166.2) × (2.0–) 2.5–4.3 (–4.9) μm. Conidiogenous cells integrated, terminal, mono- to polyblastic, producing conidia sympodially, (6.8–) 7.8–11.1 (–11.9) × (1.9–) 2.3–3.1 (–3.2) μm. Conidia pale brown to brown, smooth or slightly verrucose, thick-walled, clavate to fusiform, mostly aseptate, occasionally one-septate or with two septa, (4.4–) 5.1–7.6 (–8.8) × (3.7–) 4.3–6.3 (–7.2) μm (c; Fig. 15D–F, H). Thallic conidiation: Vegetative hyphae directly transformed into thick-walled chlamydospores, arranged in short chains, subcircular to elliptical, pale brown to brown, smooth to finely verruculose, (5.4–) 5.7–7.5 (–8.3) × (1.6–) 1.9–2.7 (–2.8) μm (d; Fig. 15G). Sexual state not observed.

###### Culture characteristics.

Colonies on MEA are slow-growing, wrinkled, with irregularly serrated edges, densely packed, and light olive green. On OA and SNA media, the colonies are black to off-white, with the aerial hyphae on SNA being fluffier. The optimal temperature is 20 °C, reaching 9–11 mm diam after 10 d. It can grow a little at 5 °C. No growth at 35 °C.

###### Additional materials examined.

**CHINA** • Jiangsu Province, Nanjing, Nanjing Forestry University, Baima Campus, fungal endophytes from *Pinus thunbergii*, May 2023, Ben Fan and Xiuyu Zhang, NF413, NF416.

###### Notes.

The genus *Rachicladosporium* currently comprises 21 accepted epithets according to Index Fungorum (https://www.indexfungorum.org), four of which have since been transferred to the genus *Cryoendolithus*. Although not all species were included in the phylogenetic analyses, a pairwise comparison of ITS sequences showed that strain NF412 differs from its closest relative, *Rachicladosporium alpinum*, by 3.28% (15 nucleotide substitutions across 458 aligned base pairs), supporting its recognition as a distinct species. Species within this genus have been reported from diverse substrates and habitats, including sooty mold communities on leaves and needles of trees and shrubs, as well as on rocks and insects (Cruywagen et al. 2015). Phylogenetic analysis shows that three new isolates of *R. pennatum*, obtained as endophytic fungi from resistant *Pinus thunbergii*, cluster within the main *Rachicladosporium* clade and are closely related to *R. alpinum* and *R. paucitum*. However, both *R. alpinum* and *R. paucitum* were isolated from rocks in the Italian Alps and produce only subhyaline to pale brown hyphae (Egidi et al. 2014; Crous et al. 2019a). Morphologically, *R. pennatum* produces both conidia and chlamydospores, whereas in the closely related species *R. alpinum*, conidia or chlamydospore-like cells have not been observed (Egidi et al. 2014).

##### ﻿ Toxicocladosporium fusiforme

Taxon classificationAnimaliaCapnodialesExtremaceae

X. Yu. Zhang, Q.Y. Zhang & B. Fan sp. nov.

1203F877-5A22-580A-A65B-7EC949B55458

C860181

Fig. 16

###### Type.

**CHINA** • Jiangsu Province, Nanjing, Nanjing Forestry University, Baima Campus, fungal endophytes from *Pinus thunbergii*, May 2023, Ben Fan and Xiuyu Zhang, NF414 (**holotype HMAS 354159**, culture ex-type CGMCC 3. 28958).

**Figure 16.**
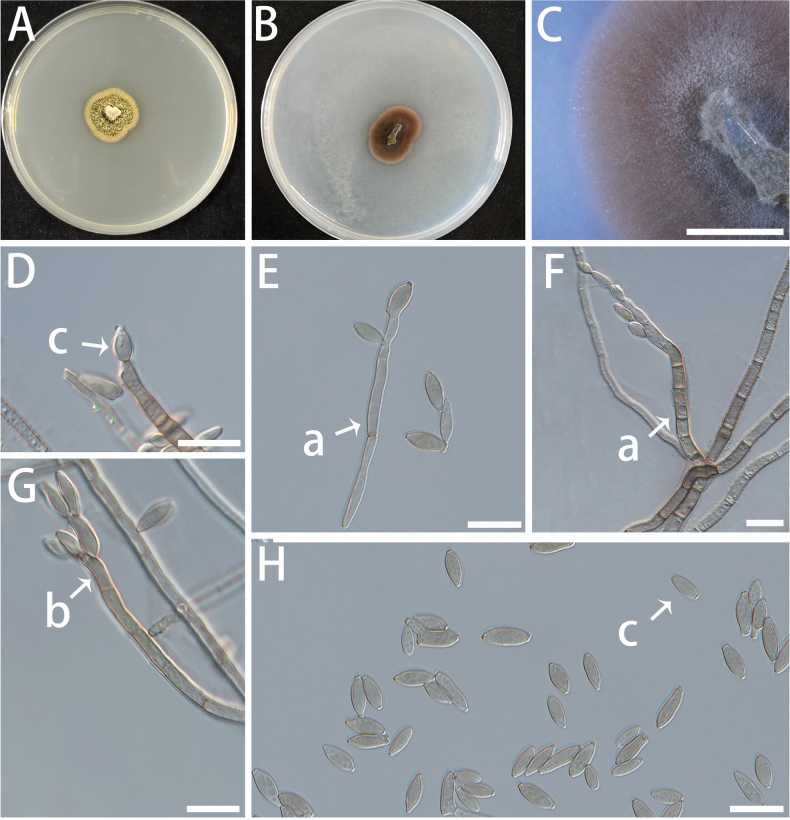
Morphological features of *Toxicocladosporium fusiforme* (isolate NF414). A, B Colonies on MEA (A) and OA (B) at 25 °C for 10 days on 90 mm Petri dish. C Colony on OA. B–E Conidiophores (a), conidiogenous cells (b), and conidia (c). Scale bars: 10 μm (B–E).

###### Etymology.

Derived from the Latin words “fusiforme”, referring to the fusiform conidia of this species.

###### Description.

Mycelium composed of septate, subhyaline to pale brown, smooth to warty, branched hyphae, 1.8–3.2 μm diam. Asexual state: Conidiophores arising from superficial mycelium, erect to sinuous, brown, unbranched, smooth or verrucous, subcylindrical, straight to flexuous, multi-septate, (21.2–) 24.4–58.7 (–78.2) × (2.1–) 2.5–3.6 (–3.9) μm. Conidiogenous cells are terminal, smooth, slightly verrucous, light brown, unseptate, subcylindrical, (4.8–) 5.2–10.5 (–11.7) × (2.1–) 2.2–3.5 (–3.7) μm. Conidia are terminal or lateral to the tips of hyphae or conidiogenous cells, smooth, transparent to light brown, without septa, fusiform, some of which are connected in series, (6.4–) 6.5–7.9 (–8.2) × (2.2–) 2.4–3.3 (–3.5) μm.

###### Culture characteristics.

Colonies on MEA surface flat to slightly raised at centre, yellow-green, fuzzy, and have intact edges; reverse dark brown. On OA, colonies are brown, fading toward the centre and edges. The optimal temperature is 25–30 °C, reaching 13–16 mm diam in 10 d. At 35 °C, the fungus shows slight growth, attaining a colony diameter of 3–5 mm within 10 days. No growth was observed at 5 °C.

###### Notes.

The genus *Toxicocladosporium* was established by Crous et al. (Crous et al. 2007b) to accommodate cladosporium-like fungi characterised by “dark, thick-walled, conidial and conidiophore septa, and lacking the typical coronate *Cladosporium* scar” (Bezerra et al. 2017). Species of *Toxicocladosporium* are widely distributed and capable of colonising a variety of substrates. Nearly all species in this genus exhibit a broad host range, and have been isolated from diverse sources including mold-infested paint and clinical specimens (Crous et al. 2009c; Crous et al. 2009d; Crous et al. 2016b). Phylogenetic analyses indicate that our new fungal strain of *T. fusiforme*, isolated from *Pinus thunbergii*, is nested within the *Toxicocladosporium* clade and closely related to *T. immaculatum*, from which it differs by 1.65% (8/484 bp) in the ITS region. Morphologically, *T. fusiforme* is distinguished by its relatively long conidiophores (21.2–78.2 vs. 12−25 × 2.5−3.5 µm in *T. immaculatum*).

##### ﻿ Zasmidium guttulatum

Taxon classificationAnimaliaCapnodialesExtremaceae

X. Yu. Zhang, Q.Y. Zhang & B. Fan sp. nov.

31DFF8FB-F2F6-51EC-BEEC-B50905401FB3

C860183

Fig. 17

###### Type.

**CHINA** • Jiangsu Province, Nanjing, Nanjing Forestry University, Baima Campus, fungal endophytes from *Pinus densiflora*, May 2023, Ben Fan and Xiuyu Zhang, NF649 (**holotype HMAS 352959**, culture ex-type CGMCC 3. 28955).

**Figure 17.**
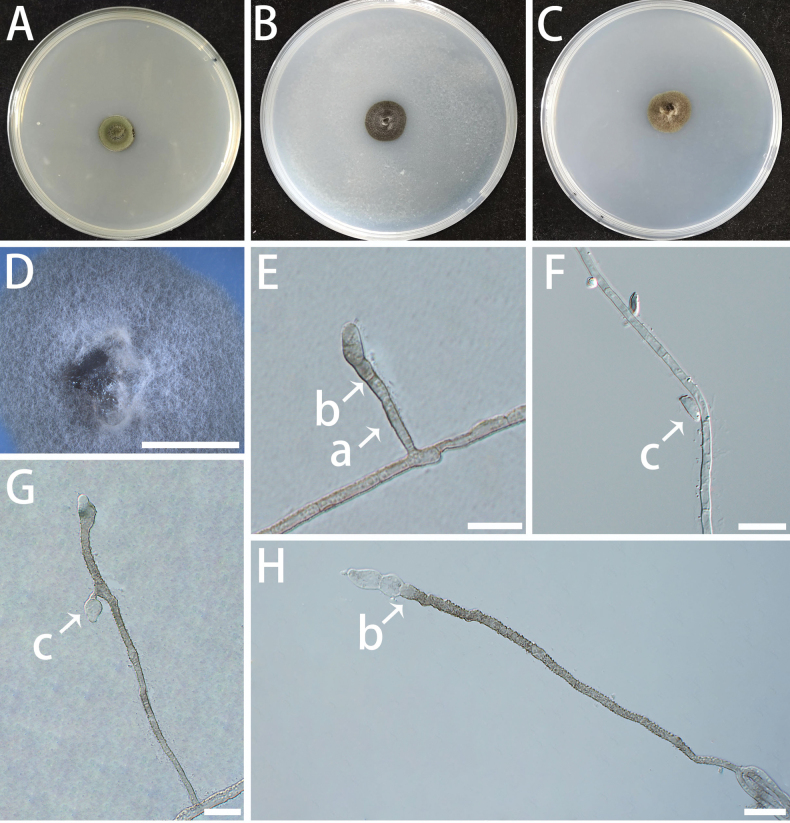
Morphological features of *Zasmidium guttulatum* (isolate NF649). A–C Colonies on MEA (A), OA (B) and SNA (C) at 25 °C for 10 days on 90 mm Petri dish. D Colony on SNA. D–H Conidiophores (a), conidiogenous cells (b), and conidia (c). Scale bars: 10 μm (D–H).

###### Etymology.

Derived from the Latin word “guttula” (a droplet), referring to the droplet-like appearance of the conidia.

###### Description.

Mycelium composed of brown, septate, branched hyphae, smooth to verruculose, 1.6–3.3 μm in diameter. Asexual state: Conidiophores erect, subcylindrical, dark brown, straight, verrucous, (19.5–) 21.8–63.0 (–80.8) × (1.4–) 1.5–2.2 (–2.5) μm. Conidiogenous cells formed by the degeneration of conidiophores or terminally, pale to brown, verrucous, cylindrical, (2.6–) 3.0–3.9 (–4.1) × (2.1–) 2.2–2.4 (–2.5) μm. Conidia terminal or lateral, drop-shaped to gourd-shaped, brown to transparent, smooth to slightly verrucous, 0–1 septate, (4.5–) 5.1–8.1 (–9.9) × (3.4–) 338–4.5 (–4.7) μm. Sexual state unknown.

###### Culture characteristics.

Colonies grow circular and flat, yellowish-green to dark olivaceous, producing droplets on MEA, with floccose aerial mycelium on SNA and OA. The optimal temperature for growth is 25 °C, reaching 11–13 mm in diameter after 10 days. No growth occurs at 5 °C or 35 °C.

###### Additional material examined.

**CHINA** • Jiangsu Province, Nanjing, Nanjing Forestry University, Baima Campus, fungal endophytes from *Pinus densiflora*, May 2023, Ben Fan and Xiuyu Zhang, NF648.

###### Notes.

Two isolates, NF649 and NF849 (*Zasmidium guttulatum*), obtained from endophytic fungi, formed a clade closely related to *Z. pearceae*. However, they differ morphologically. Colonies of NF649 produce viscous droplets on the surface and have a regular margin, in contrast to the dry surface and irregular margin of *Z. pearceae* colonies. In addition, the conidial morphology is distinct, and NF649 produces guttulate, drop-shaped conidia, while those of *Z. pearceae* are elongate and cylindrical (Tan et al. 2022).

##### ﻿ Zasmidium longisporum

Taxon classificationAnimaliaCapnodialesExtremaceae

X. Yu. Zhang, Q.Y. Zhang & B. Fan sp. nov.

D67F9C5A-8148-598E-9F88-684E343F1FB9

C860187

Fig. 18

###### Type.

**CHINA** • Jiangsu Province, Nanjing, Nanjing Forestry University, Baima Campus, fungal endophytes from *Pinus densiflora*, May 2023, Ben Fan and Xiuyu Zhang, NF622 (**holotype HMAS 352958**, culture ex-type CGMCC 3. 28954).

###### Etymology.

Derived from the Latin words “longus” and “spora”, the species epithet refers to the elongated conidia produced by the fungus.

###### Description.

**Figure 18.**
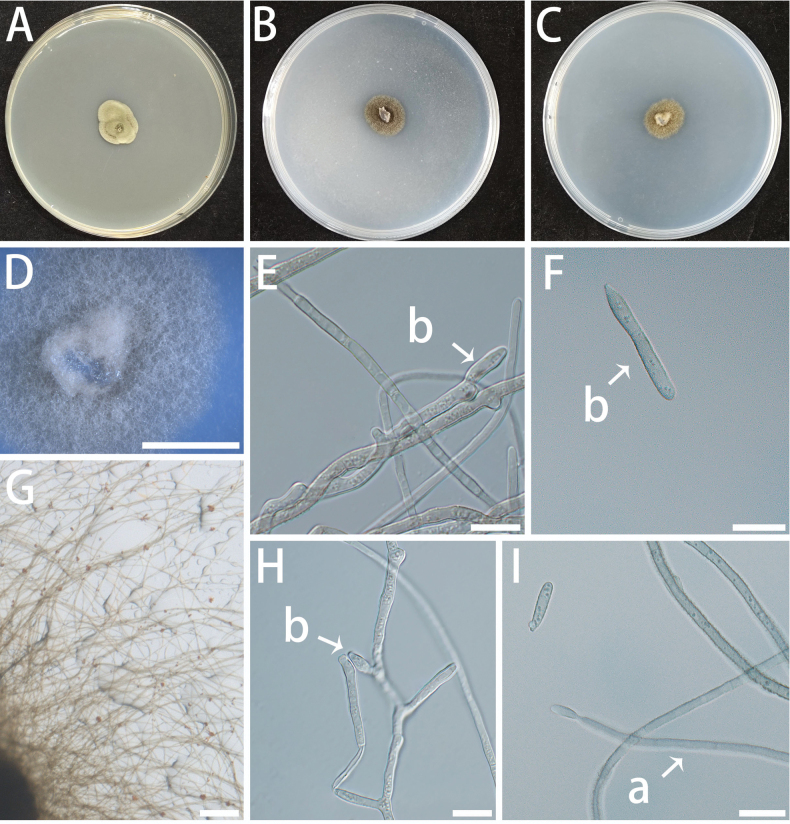
Morphological features of *Zasmidium longisporum* (NF622). A–C Colonies on MEA (A), OA (B) and SNA (C) at 25 °C for 10 days on 90 mm Petri dish. D Colony on SNA. G Hyphal morphology on SNA. E, F, H, I Conidiophores (a) and conidia (b). Scale bars: 5 mm (D);100 μm (G); 10 μm (E, F, H, I)

Mycelium composed of branched, septate, pale brown, verruculose hyphae, 1.5–3 μm diam. Numerous orange-coloured granules are observed among the hyphae (Fig. 18G). Asexual state: Conidiophores erect or curved, cylindrical, hyaline to pale brown, aseptate, smooth, (17.2–) 30.9–58.1 (–79.5) × (1.9–) 2.3–3.1 (–3.7) μm. Conidiogenous cells absent; conidia produced directly from the sides or apices of hyphae, hyaline, subcylindrical with a slightly narrowed base, slightly verruculose, mostly aseptate, occasionally with one septum, (6.1–) 7.7–16.2 (–21.2) × (2.3–) 2.7–3.3 (–3.6) μm. Sexual state unknown.

###### Culture characteristics.

Colonies on MEA with irregular margins and slightly raised centres, bearing abundant aerial mycelium, yellowish-green to cream-coloured. On SNA and OA, aerial mycelium is sparse; colonies appear dark olivaceous on OA and deep yellowish-green on SNA. The optimal temperature is 25 °C, with colonies reaching 12–14 mm in diameter in 10 days. No growth at 5 °C or 35 °C.

###### Additional material examined.

**CHINA** • Jiangsu Province, Nanjing, Nanjing Forestry University, Baima Campus, fungal endophytes from *Pinus densiflora*, May 2023, Ben Fan and Xiuyu Zhang, NF822.

###### Notes.

Two isolates, NF622 and NF622-2, were identified as *Zasmidium longisporum*, a species phylogenetically related to *Z. fructicola*. The two taxa can be distinguished primarily by conidial size: NF622 produces significantly larger conidia, measuring up to 21.2 μm, compared to those of *Z. fructicola*, which reach a maximum length of 12 μm. Additionally, NF622 exhibits slower growth, achieving a colony diameter of 12–14 mm after 10 days, whereas *Z. fructicola* grows more rapidly, reaching 40 mm in diameter within 14 days. Ecologically, NF622 was isolated as an endophytic fungus from pine trees, while *Z. fructicola* is a known pathogen of citrus fruit (Huang 2015).

## ﻿Discussion

The diversity and ecological roles of endophytic fungi, particularly in coniferous hosts, remain significantly underexplored. This study substantially expands this knowledge by proposing three novel genera (*Helianthoconium*, *Botryoconidia*, *Longisporomyces*) and nine novel species (*Botryoconidia globosus*, *Longisporomyces filisporum*, *Neocatenulostroma endophyticum*, *Rachicladosporium pennatum*, *Helianthoconium helianthosporum*, *Sphaerulina nanjingensis*, *Toxicocladosporium fusiforme*, *Zasmidium guttulatum*, and *Zasmidium longisporum)* isolated as endophytes from *Pinus* in China, based on robust morphological and multi-locus phylogenetic evidence. These unique and new lineages at the genus or species level within the established families represent novel taxa of the order *Capnodiales* s. lat. proposed in this study.

The family *Dissoconiaceae*, established in 2009 to accommodate *Dissoconium* species, which possess *Mycosphaerella*-like teleomorphs, forms a distinct clade that clusters between the families *Teratosphaeriaceae* and *Schizothyriaceae* (Crous et al. 2009d). Originally, *Dissoconiaceae* was established to accommodate *Dissoconium* and *Ramichloridium*, both reported as hyperparasites on powdery mildews on various hosts (Hijwegen and Buchenauer 1984). Subsequently, this family was expanded to incorporate four additional genera: *Globoramichloridium* Y. Marin & Crous, *Paradissoconium* Crous & Boers, *Pseudoveronaea* Crous & Batzer, and *Uwebraunia* Crous & M.J. Wingf. (JC et al. 2005; Sun et al. 2007; Díaz Arias et al. 2010; Gleason et al. 2011). In this study, the endophytic fungi isolated from pine trees in this study formed a highly supported, distinct clade sister to, but clearly separated from, known genera within *Dissoconiaceae* (Fig. 2). Morphologically, the short conidiophores and spherical conidia observed in our isolates contrasted with those typical of other *Dissoconiaceae* members. The establishment of the genus *Helianthoconium* is supported by multiple lines of evidence. Phylogenetically, it constitutes a well-supported, independent lineage within the *Dissoconiaceae*, a finding corroborated by significant genetic divergence in the ITS region. Morphologically, it possesses a unique autapomorphy in the form of sunflower seed-shaped conidia, which is not observed in other genera of the family. Therefore, we propose the establishment of *Helianthoconium* as a novel genus within the *Dissoconiaceae*, with *Helianthoconium helianthosporum* as its generic type. Our divergence time analysis further supports its distinctness, indicating an ancient origin (90 Mya, Fig. 9), which places it among early diverging lineages potentially related to *Mycosphaerellaceae* ancestors. Together, the phylogenetic isolation, morphological uniqueness, and deep divergence provide compelling evidence for establishing *Helianthoconium* within *Dissoconiaceae*.

The majority of the species within *Extremaceae* are known to inhabit extreme environments, such as Antarctic rocks and craters (Laichmanova 2023). In this study, two strains isolated from the endophytic fungi of pine trees were assigned to this family and formed a well-supported clade. This clade was phylogenetically distant from the type genus *Extremus* and other described genera in *Extremaceae*. Morphologically, the conidial shape of our isolates also differed from known morphotypes within this family. Conidia of other genera in *Extremaceae* are solitary or in chains, whereas conidia of our isolates are wrinkled and form grape-like clusters. Consequently, we propose the establishment of *Botryoconidia* as a new genus within the *Extremaceae*, with *Botryoconidia globosus* as its representative species. Crucially, divergence time estimation revealed *Botryoconidia* (167.03 Mya, Fig. 9) as one of the earliest-diverging lineages within *Capnodiales*. This deep evolutionary divergence and phylogenetic distinctness support the recognition of *Botryoconidia* as a novel genus and extend the known ecological range of *Extremaceae* beyond extreme environments to include conifer endophytes.

The *Mycosphaerellaceae* is the largest family within the order *Mycosphaerellales*, comprising over 100 genera and approximately 1,000 species (Bakhshi et al. 2015; Videira et al. 2017; Crous et al. 2020b; Rajeshkumar et al. 2021). Members of this family are cosmopolitan fungi exhibiting diverse ecological roles, ranging from plant pathogenic to endophytic, saprobic, and even hyperparasitic lifestyles, and are associated with a wide variety of hosts and substrates. Within the *Mycosphaerellaceae*, our isolates formed a stable, well-supported clade (Fig. 4), which showed no close affinity to any described genus and exhibited a unique combination of morphological features, particularly exceptionally long conidia. In this study, a novel monotypic genus, *Longisporomyces*, has been proposed in this family, with the novel species *Longisporomyces filisporum*. Divergence time analysis indicated its relatively recent emergence (22.81 Mya, Fig. 9), comparable to *Xenosonderhenioides* C. Nakash., Videira & Crous, positioning it among later diverging lineages in *Mycosphaerellaceae*. This clear phylogenetic distinction coupled with distinctive morphological traits and a defined divergence point, supports its recognition as a new monotypic genus.​​

In this work, divergence times were estimated using a fossil-calibrated phylogeny based on the methodology and calibration points established in Hongsanan et al. (2020). These estimates provide key independent evidence supporting the distinct status and phylogenetic placement of our proposed novel genera. The ancient divergence of *Helianthoconium* (90 Mya) and *Botryoconidiа* (167.03 Mya) confirms their position as deeply rooted, early-splitting lineages. The deep evolutionary history of these lineages may suggest potentially ancient origin of endophytism in *Capnodiales*, although this hypothesis must be treated with caution due to the limited taxon sampling in the present study. Conversely, the relatively recent divergence of Longisporomyces (22.8 Mya) is consistent with the hypothesis of continued diversification within *Mycosphaerellaceae* during the Neogene. Future studies with broader sampling are needed to test these preliminary inferences. Additionally, the considerable temporal gaps between the novel genera and their closest known relatives further substantiates their taxonomic novelty.

The genus *Rachicladosporium* is extensively studied within the family *Cladosporiaceae*. The genus was initially established to accommodate the strains isolated from leaf spots on *Luculia* species in New Zealand, with *Rachicladosporium luculiae* Crous, U. Braun & C.F. Hill (Crous et al. 2007b; Piątek et al. 2023) as the type species. Subsequent research has shown that species within this genus occur in a diverse range of substrates, habitats, and environments, including plant leaves and needles, twigs, black mold on baobab trees, rocks, and insects (Crous 2009; Egidi et al. 2014; Cruywagen et al. 2015). Among these studies, Qadri et al. (2014) also detect unclassified *Rachicladosporium* species among the endophytes of various *Pinus* species in the Western Himalayas. Similarly, in this study, three endophytic fungi from pine trees in China were identified as a novel species of *Rachicladosporium*. The name *Rachicladosporium pennatum* is proposed here for the new species.

The genus *Toxicocladosporium* is another important genus within the family *Cladosporiaceae*. This genus is predominantly associated with plant materials as epiphyte, saprobe, or phytopathogen, and is also reported from unusual substrates or hosts (Crous et al. 2007b; Bensch et al. 2012). Some species have been recovered from environments such as moldy paint and a human clinical specimen—for example, *T. irritans* and *T. hominis* from bronchoalveolar lavage fluid (Crous et al. 2007b; Crous et al. 2016b). However, its reported occurrence as an endophyte remains limited. To date, only Bezerra et al. (2017) have documented *Toxicocladosporium* among fungal endophytes in cacti, describing two new species: *T. cacti* and *T. immaculatum*. In this study, a new species, *T. fusiforme*, formed a well-supported monophyletic lineage and was closely related to *T. immaculatum*. Notably, the divergence time analysis suggests that *T. fusiforme* represents the earliest diverging species within *Cladosporiaceae*, split approximately 85.68 Mya.

*Neocatenulostroma* is a small genus of *Teratosphaeriaceae*. Currently, it comprises only five described species, all of which are plant pathogenic fungi associated with pine shoot blight diseases, including on *Pinus mugo* Turra and *Pinus sylvestris* L. (Markovskaja et al. 2016). Here, two endophytic fungal strains were isolated from healthy pine trees in China and identified as a novel species within *Neocatenulostroma*. This novel species is named *N. endophyticum*, which exhibits melanized, black yeast-like morphological features similar to those found in other species of this genus. This finding expanded the known lifestyle diversity of the *Neocatenulostroma* species beyond pathogenicity, suggesting a more varied ecological role for the genus. However, it cannot be excluded that *N. endophyticum* have the potential to act as a pathogen on specific host plants, a possibility that requires further investigation.

Briefly, the discovery of multiple novel genera and species as *Pinus* endophytes significantly enhances our understanding of the *Capnodiales*--*Pinus* association. Previous studies on *Capnodiales* in conifers have primarily focused on pathogenic taxa (e.g. *Mycosphaerella*, *Dothistroma* and *Neocatenulostroma*, which cause needle blights). Our findings reveal a previously undocumented endophytic diversity of *Capnodiales* within *Pinus* tissues. The discovery of *Neocatenulostroma endophyticum* as an endophyte is particularly noteworthy, as this genus was previously recognised only as a pathogen of *Pinus*. This demonstrates the dual ecological potential (endophytic/latent pathogen) within a genus hitherto defined by its pathogenicity. Similarly, families like *Dissoconiaceae* (known as hyperparasites) and *Extremaceae* (typically extremophiles) are now also confirmed to harbour endophytic representatives in conifers.

Furthermore, the endophytic lifestyle of the new taxa we proposed such as *Helianthoconium (Dissoconiaceae)*, *Botryoconidia (Extremaceae)*, and *Longisporomyces (Mycosphaerellaceae)* inside *Pinus* represents a novel ecological role for these lineages/families.​ The ​remarkable ancient divergence of *Botryoconidia* (167.03 Mya) suggests an evolutionary history potentially intertwined with conifers during the Mesozoic era, which may confer adaptations reflected in its current endophytic niche. The presence of these diverse novel endophytes underscores that the ecological interactions between *Capnodiales* and *Pinus* are more complex and involves greater cryptic diversity than previously recognized, extending well beyond known pathogens and epiphytes. Additionally, the discovery of new species such as *Rachicladosporium pennatum* and *Toxicocladosporium fusiforme* further demonstrates the wide environmental adaptability of the *Cladosporiaceae*.​

This study expands our understanding of the ecological diversity and taxonomic framework of *Capnodiales* s. lat., particularly regarding its association with conifer endophytes. The diversity of endophytic fungi within this order is likely still largely unexplored. Future research should prioritise elucidating the functional roles of these novel endophytes, their host specificity within Pinus and other conifers, as well as their potential applications.

## ﻿Conclusion

Based on an analysis of more than 800 endophytic fungal isolates from healthy *Pinus* shoots in China, we have introduced three novel genera and nine novel species within the order *Capnodiales* s. lat.. Phylogenetic reconstructions using a multi-locus dataset strongly support the placement of the three novel genera within *Dissoconiaceae*, *Extremaceae*, and *Mycosphaerellaceae*. Furthermore, fossil-calibrated divergence time estimation indicates a Middle Triassic origin (241.9 Mya) for *Capnodiales* s. lat., with major subclades diversifying during the Jurassic. These findings provide a valuable evolutionary perspective on the diversification of this group. Overall, our findings significantly expand the current taxonomic framework of *Capnodiales* s. lat., highlight the largely underexplored diversity of conifer endophytes, and lay a foundation for future studies addressing their functional roles, host associations, and potential applications in forestry and biotechnology.

## Supplementary Material

XML Treatment for Helianthoconium

XML Treatment for Helianthoconium helianthosporum

XML Treatment for Botryoconidia

XML Treatment for Botryoconidia globosus

XML Treatment for Longisporomyces

XML Treatment for Longisporomyces filisporum

XML Treatment for Neocatenulostroma endophyticum

XML Treatment for Sphaerulina nanjingensis

XML Treatment for Rachicladosporium pennatum

XML Treatment for Toxicocladosporium fusiforme

XML Treatment for Zasmidium guttulatum

XML Treatment for Zasmidium longisporum

## ﻿Additional information

### Use of AI

No use of AI was reported.

### Adherence to national and international regulations

All the fungal strains used in this study have been legally obtained, respecting the Convention on Biological Diversity (Rio Convention).

### Author ORCIDs

Xiu-Yu Zhang https://orcid.org/0000-0002-8678-8446

Qiu-Yue Zhang https://orcid.org/0000-0001-9458-3566

De-Wei Li https://orcid.org/0000-0002-2788-7938

Jing-Zu Sun https://orcid.org/0000-0003-1893-1869

Ben Fan https://orcid.org/0000-0001-8532-1281

### Data availability

All of the data that support the findings of this study are available in the main text or Supplementary Information.

## Supplementary materials

Supplementary material 1 Cultures examined in this study and their GenBank accession numbers Data type xlsx https://binary.pensoft.net/file/1489731This dataset is made available under the Open Database License (http://opendatacommons.org/licenses/odbl/1.0/). The Open Database License (ODbL) is a license agreement intended to allow users to freely share, modify, and use this Dataset while maintaining this same freedom for others, provided that the original source and author(s) are credited.Xiu-Yu Zhang, Qiu-Yue Zhang, De-Wei Li, Jing-Zu Sun, Ben Fan

Supplementary material 2 Linked data table template for primary biodiversity data Data type xlsx https://binary.pensoft.net/file/1489732This dataset is made available under the Open Database License (http://opendatacommons.org/licenses/odbl/1.0/). The Open Database License (ODbL) is a license agreement intended to allow users to freely share, modify, and use this Dataset while maintaining this same freedom for others, provided that the original source and author(s) are credited.Xiu-Yu Zhang, Qiu-Yue Zhang, De-Wei Li, Jing-Zu Sun, Ben Fan

Supplementary material 3 *Capnodiales* LSU-ITS-RPB2-TEF alignment Data type fas https://binary.pensoft.net/file/1489733This dataset is made available under the Open Database License (http://opendatacommons.org/licenses/odbl/1.0/). The Open Database License (ODbL) is a license agreement intended to allow users to freely share, modify, and use this Dataset while maintaining this same freedom for others, provided that the original source and author(s) are credited.Xiu-Yu Zhang, Qiu-Yue Zhang, De-Wei Li, Jing-Zu Sun, Ben Fan

Supplementary material 4 *Dissoconiaceae*ITS-LSU alignment Data type fas https://binary.pensoft.net/file/1489734This dataset is made available under the Open Database License (http://opendatacommons.org/licenses/odbl/1.0/). The Open Database License (ODbL) is a license agreement intended to allow users to freely share, modify, and use this Dataset while maintaining this same freedom for others, provided that the original source and author(s) are credited.Xiu-Yu Zhang, Qiu-Yue Zhang, De-Wei Li, Jing-Zu Sun, Ben Fan

Supplementary material 5 *Extremaceae*ITS-LSU alignment Data type fas https://binary.pensoft.net/file/1489735This dataset is made available under the Open Database License (http://opendatacommons.org/licenses/odbl/1.0/). The Open Database License (ODbL) is a license agreement intended to allow users to freely share, modify, and use this Dataset while maintaining this same freedom for others, provided that the original source and author(s) are credited.Xiu-Yu Zhang, Qiu-Yue Zhang, De-Wei Li, Jing-Zu Sun, Ben Fan

Supplementary material 6 *Mycosphaerellaceae*ITS-LSU-RPB2 alignment Data type fas https://binary.pensoft.net/file/1489736This dataset is made available under the Open Database License (http://opendatacommons.org/licenses/odbl/1.0/). The Open Database License (ODbL) is a license agreement intended to allow users to freely share, modify, and use this Dataset while maintaining this same freedom for others, provided that the original source and author(s) are credited.Xiu-Yu Zhang, Qiu-Yue Zhang, De-Wei Li, Jing-Zu Sun, Ben Fan

Supplementary material 7 *Cladosporiaceae*ITS-LSU-RPB2 alignment Data type fas https://binary.pensoft.net/file/1489737This dataset is made available under the Open Database License (http://opendatacommons.org/licenses/odbl/1.0/). The Open Database License (ODbL) is a license agreement intended to allow users to freely share, modify, and use this Dataset while maintaining this same freedom for others, provided that the original source and author(s) are credited.Xiu-Yu Zhang, Qiu-Yue Zhang, De-Wei Li, Jing-Zu Sun, Ben Fan

Supplementary material 8 *Sphaerulina*ITS-LSU-RPB2-TUB-TEF alignment Data type fas https://binary.pensoft.net/file/1489738This dataset is made available under the Open Database License (http://opendatacommons.org/licenses/odbl/1.0/). The Open Database License (ODbL) is a license agreement intended to allow users to freely share, modify, and use this Dataset while maintaining this same freedom for others, provided that the original source and author(s) are credited.Xiu-Yu Zhang, Qiu-Yue Zhang, De-Wei Li, Jing-Zu Sun, Ben Fan

Supplementary material 9 *Teratosphaeriaceae* LSU-ITS-ACT-CAL-RPB2-TEF alignment Data type fas https://binary.pensoft.net/file/1489739This dataset is made available under the Open Database License (http://opendatacommons.org/licenses/odbl/1.0/). The Open Database License (ODbL) is a license agreement intended to allow users to freely share, modify, and use this Dataset while maintaining this same freedom for others, provided that the original source and author(s) are credited.Xiu-Yu Zhang, Qiu-Yue Zhang, De-Wei Li, Jing-Zu Sun, Ben Fan

Supplementary material 10 *Zasmidium*ITS-LSU-RPB2 alignment Data type fas https://binary.pensoft.net/file/1489740This dataset is made available under the Open Database License (http://opendatacommons.org/licenses/odbl/1.0/). The Open Database License (ODbL) is a license agreement intended to allow users to freely share, modify, and use this Dataset while maintaining this same freedom for others, provided that the original source and author(s) are credited.Xiu-Yu Zhang, Qiu-Yue Zhang, De-Wei Li, Jing-Zu Sun, Ben Fan

Supplementary material 11 *Capnodiales* divergen ITS-LSU-RPB2 alignment Data type fas https://binary.pensoft.net/file/1489741This dataset is made available under the Open Database License (http://opendatacommons.org/licenses/odbl/1.0/). The Open Database License (ODbL) is a license agreement intended to allow users to freely share, modify, and use this Dataset while maintaining this same freedom for others, provided that the original source and author(s) are credited.Xiu-Yu Zhang, Qiu-Yue Zhang, De-Wei Li, Jing-Zu Sun, Ben Fan
